# Regulation of mitochondrial complex III activity and assembly by TRAP1 in cancer cells

**DOI:** 10.1186/s12935-022-02788-4

**Published:** 2022-12-12

**Authors:** Danilo Swann Matassa, Daniela Criscuolo, Rosario Avolio, Ilenia Agliarulo, Daniela Sarnataro, Consiglia Pacelli, Rosella Scrima, Alessandra Colamatteo, Giuseppe Matarese, Nazzareno Capitanio, Matteo Landriscina, Franca Esposito

**Affiliations:** 1 Department of Molecular Medicine and Medical Biotechnology, University of Naples Federico II, 80131 Naples, Italy; 2 Institute of Biochemistry and Cellular Biology, National Research Council of Italy (CNR), 80131 Naples, Italy; 3 Department of Clinical and Experimental Medicine, University of Foggia, 71122 Foggia, Italy; 4 Institute Experimental Endocrinology and Oncology “Gaetano Salvatore”, National Research Council (IEOS-CNR), 80131 Naples, Italy; 5 Department of Medical and Surgical Science, University of Foggia, 71122 Foggia, Italy; 6 Laboratory of Pre-Clinical and Translational Research, IRCCS, Referral Cancer Center of Basilicata, 85028 Rionero in Vulture, Italy

**Keywords:** TRAP1, Respiratory complex III, Ovarian cancer, Platinum resistance

## Abstract

**Background:**

Metabolic reprogramming is an important issue in tumor biology. A recently-identified actor in this regard is the molecular chaperone TRAP1, that is considered an oncogene in several cancers for its high expression but an oncosuppressor in others with predominant oxidative metabolism. TRAP1 is mainly localized in mitochondria, where it interacts with respiratory complexes, although alternative localizations have been described, particularly on the endoplasmic reticulum, where it interacts with the translational machinery with relevant roles in protein synthesis regulation.

**Results:**

Herein we show that, inside mitochondria, TRAP1 binds the complex III core component UQCRC2 and regulates complex III activity. This decreases respiration rate during basal conditions but allows sustained oxidative phosphorylation when glucose is limiting, a condition in which the direct TRAP1-UQCRC2 binding is disrupted, but not TRAP1-complex III binding. Interestingly, several complex III components and assembly factors show an inverse correlation with survival and response to platinum-based therapy in high grade serous ovarian cancers, where TRAP1 inversely correlates with stage and grade and directly correlates with survival. Accordingly, drug-resistant ovarian cancer cells show high levels of complex III components and high sensitivity to complex III inhibitory drug antimycin A.

**Conclusions:**

These results shed new light on the molecular mechanisms involved in TRAP1-dependent regulation of cancer cell metabolism and point out a potential novel target for metabolic therapy in ovarian cancer.

**Supplementary Information:**

The online version contains supplementary material available at 10.1186/s12935-022-02788-4.

## Background

TRAP1 is a multifaced protein, since initially described as a chaperone for the retinoblastoma protein during mitosis and after heat shock [1], as a TNF-Receptor associated protein [2] and as a factor stabilizing CypD, which prevents permeability transition pore opening and thus apoptosis [3]. However, in the last few years TRAP1 has emerged as a critical regulator of mitochondrial respiration, through the direct binding to respiratory complexes [4], and as a regulator of cytoplasmic protein synthesis, through the binding to ribosomes and translation factors [5]. The regulation of cancer cell metabolism by TRAP1 appears to have contextual effects on cancer onset and progression, thus favoring the oncogenic phenotype in glycolytic tumors, while being negatively selected in tumors mostly relying on oxidative metabolism [6]. At first, three different groups independently demonstrated that TRAP1 had a significant and direct impact on mitochondrial respiration. Yoshida and colleagues showed that TRAP1 deficiency in immortalized mouse fibroblasts and in human tumor cells promotes an increase in mitochondrial respiration and fatty acid oxidation, and results in cellular accumulation of tricarboxylic acid cycle intermediates, ATP and reactive oxygen species; a basis for a mechanistic model for these regulations was provided by the finding that TRAP1 binds and consequently inhibits phosphorylation of mitochondrial cSrc, which is able to stimulate respiratory chain complex IV [7]. Sciacovelli and colleagues also discovered that TRAP1 silencing induces an increase in respiration, but they showed that this is due to a direct binding and inhibition of activity of succinate dehydrogenase, the complex II of the respiratory chain [8]. Accordingly, Chae and collaborators identified a direct binding between TRAP1 and succinate dehydrogenase; however they postulated that this binding stabilizes the electron transport chain complex II, maintaining cellular respiration under low-nutrient conditions [9]. Also, Park et al. [10] showed that cooperative interplay between the mitochondrial chaperone TRAP1 and the major mitochondria deacetylase sirtuin-3 in glioma stem cells increases mitochondrial respiratory capacity and reduces production of reactive oxygen species, facilitating adaptation to reduced nutrient availability. Furthermore, TRAP1 knockout in mice induces a global upregulation of oxidative phosphorylation and glycolysis transcriptomes, causing deregulated mitochondrial respiration, oxidative stress, impaired cell proliferation, and a switch to glycolytic metabolism in vivo [11]. TRAP1 −/− mice, however, were viable and displayed reduced incidence of age-related metabolic pathologies [11]; conversely, TRAP1 gene ablation in zebrafish delays embryogenesis while increasing mitochondrial respiration of fish larvae [12]. When deeply looking in cell lines at the molecular mechanisms underlying such complex regulations, it was found that the disruption of the gene for TRAP1 induced the anaplerotic utilization of glutamine metabolism to replenish tricarboxylic acid cycle intermediates, and that TRAP1 aggregates in tetrameric form in response to both increased and decreased oxidative phosphorylation [13]. These recent results provide key indications for future studies, but still fail to draw a mechanistic model for TRAP1 functions within the organelle. In the present work, we identify the respiratory complex III core component UQCRC2 as a novel TRAP1 binding partner, and demonstrate that such binding affects complex III activity at the steady state, affecting its availability for compensatory activation during metabolic stress induced by glucose deprivation. In addition, we also show that other complex III components are upregulated in the late stages of human high grade serous ovarian cancers (HGSOCs), in which TRAP1 is downmodulated, and identify the assembly factor TTC19 as a novel biomarker of potential clinical interest in this tumor type.

## Results

### TRAP1 binds to and regulates the activity of respiratory complex III

**Fig. 1:**
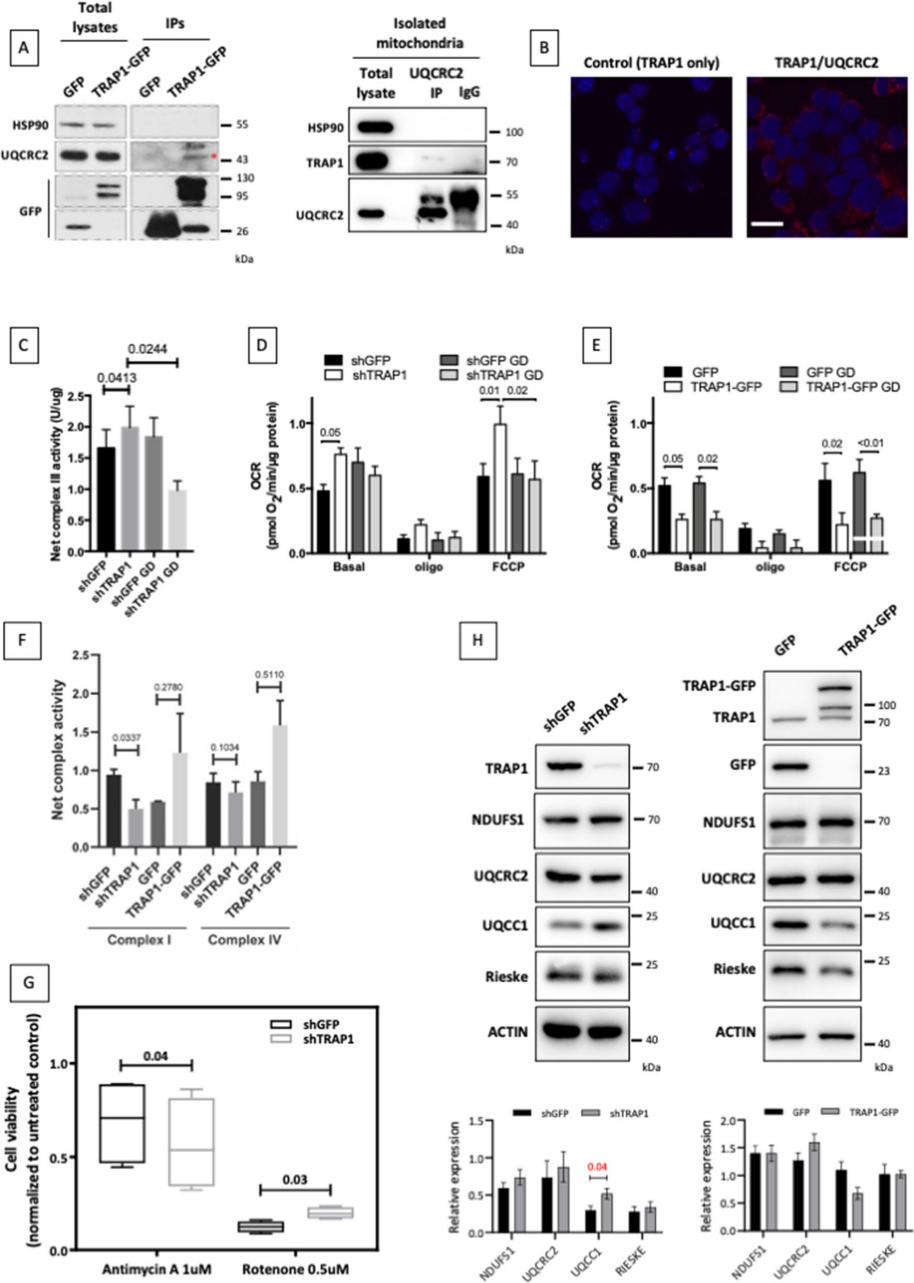
TRAP1 binds and regulates activity of complex III. **A** Co-immunoprecipitation of unfused GFP and TRAP1-GFP with UQCRC2 performed in HeLa cells following 24 h of tetracycline-mediated induction of GFP and TRAP1-GFP (left): total lysates were incubated with GFP-trap beads to isolate the proteins and the resulting samples were immunoblotted with indicated antibodies, and co-immunoprecipitation of endogenous UQCRC2 and TRAP1 (right): mitochondrial extract from HeLa cells has been incubated with anti-UQCRC2 antibody, captured with dynabeads and immunoblotted with indicated antibodies. **B** Representative image of PLA showing the interaction of TRAP1 with UQCRC2 in HCT116 cells. Positive signals of interaction are shown as red dots, nuclei are stained with DAPI (blue). Negative control has been obtained by hybridizing cells with TRAP1 antibody only. Scale bar: 50 µm. **C** Viable mitochondria isolated from inducible shGFP (control) and shTRAP1 HeLa cells quantified and mixed with a buffer containing cytocrome c. The absorbance of cytochrome c reduced from the isolated mitochondria has been measured at 550 nm. Number above bars indicate the two-tailed *p* value based on the Student’s t-test (n = 3). **D**, **E** Oxygen consumption rates (OCR) in HeLa cells following induction of shGFP/shTRAP1 for 72 h (**A**) or GFP/TRAP1-GFP for 48 h (**B**), assayed by the SeaHorse platform as described under “Metabolic analyses” section. Basal, resting OCR; Oligo, OCR measured after the addition of the ATP synthase inhibitor oligomycin also referred as proton leak; FCCP, OCR measured after the addition of the uncoupler FCCP eliciting the maximal respiratory capacity. The OCR was corrected for the residual OCR measured after the addition of the rotenone and Antimycin A inhibitors. Before metabolic assay procedures, cells have been cultured in standard culture medium (4.5 g/L glucose) or in low glucose (1 g/L) for 18 h (GD: glucose deprivation) (**E**). **F** Complex I and complex IV activity assays were performed spectrophotometrically in cell homogenates as detailed under “Complex I and complex IV activity assay” section. Data are normalized to the citrate synthase activity and expressed as mean ± SEM of three independent experiments. Numbers indicate the statistical significance (*p* value) obtained by the Student’s t-test; the citrate synthase activity did not show statistically significant change in all the cell samples tested. **G** Viability assays performed in shGFP/shTRAP1 HeLa cells following treatment with Antimycin A and Rotenone for 48 h. Data are expressed as mean ± SEM of four independent experiments, with technical triplicates each. Numbers indicate the statistical significance (*p* value) obtained by the Student’s t-test. **H** Total lysates obtained from HeLa cells after tetracycline-mediated induction of control GFP-directed (shGFP) or TRAP1-directed (shTRAP1) shRNA (72 h) or of unfused GFP or the TRAP1-GFP fusion protein (24 h) were separated by SDS-PAGE and immunoblotted with the indicated antibodies. Images are representative of four (GFP/TRAP1-GFP) or five (shGFP/shTRAP1) independent experiments. The bar graph shows densitometric band quantification, represented as mean ± SEM. Numbers above bars indicate the statistical significance (*p* value) obtained by the Student’s t-test

It has been previously shown that TRAP1 directly binds complex II of the mitochondrial respiratory chain [8, 9], modulating its stability/activity, and indirectly regulates complex IV [7]. A previously unpublished mass spectrometry analysis, which yielded several already validated TRAP1 partners [5, 14, 15], suggested that TRAP1 also binds UQCRC2, a component of complex III. Recently published proteomic analyses also supported the TRAP1-UQCRC2 partnership [16]. We have validated this interaction by both GFP-trap in inducible TRAP1-GFP HeLa cells and by immunoprecipitation of endogenous UQCRC2 from isolated HeLa mitochondria (Fig. 1A), and immunoprecipitation of a flag-UQCRC2 transfected in HeLa cells (Additional file 1: Fig. S1A). We further supported these data by proximity ligation assay (PLA) in both HCT116 colorectal carcinoma cells (Fig. 1B) and HeLa cells (Additional file 1: Fig. S1B). Notably, proximity ligation between TRAP1 and another complex III component, the catalytic subunit UQCRFS1/Rieske protein, yielded negative results, supporting the specificity of the binding between TRAP1 and UQCRC2 (Additional file 1: Fig. S1B). These findings suggest that TRAP1 regulation of the respiratory chain activity could also rely on the modulation of complex III activity.

The effects of TRAP1 on mitochondrial respiration are still controversial: TRAP1 silencing increases oxygen consumption in SAOS-2 osteosarcoma cells, PEA1 ovarian cancer cells, HCT116 colorectal carcinoma cells and mouse fibroblasts [7, 8, 17, 18]; however, TRAP1 silencing or treatment with the mitochondria-directed HSP90 inhibitor Gamitrinibs reduces oxygen consumption and ATP production in PC3 prostate cancer cells and in LN229 glioblastoma cells, although in the specific metabolic context of low glucose availability [9]. To shed light on this complex scenario, as a preliminary approach we first analyzed the response of TRAP1 knock-down cells to a decreased glucose availability through the monitoring of AMPK activation over time and identified a significant AMPK phosphorylation after 4 h of glucose withdrawal (referred to as "low glucose” hereafter), specifically in TRAP1-expressing cells (Additional file 1: Fig. S1C). Therefore, the activity of the complex III upon TRAP1 silencing was compared in standard cell culture conditions (4.5 g/L glucose) and after culturing cells in low glucose (1 g/L) medium for 4 h. Our results showed that shRNA-mediated TRAP1 silencing in HeLa cells induces a slight increase of complex III activity, as measured by an in vitro assay performed on isolated functional mitochondria (Fig. 1C). However, upon glucose withdrawal, only TRAP1 expressing cells preserve the complex III activity, whereas shTRAP1 cells dramatically lose this ability (Fig. 1C). This finding extends to complex III the direct contribution of TRAP1 to mitochondrial metabolism, which so far involved complex II, through direct interaction, besides the indirect regulation of complex IV [4]. In order to support these data in living cells, we performed a metabolic analysis using the Seahorse technology on both shRNA-mediated TRAP1 silenced HeLa cells and on TRAP1-GFP overexpressing HeLa cells. As a result, we found that TRAP1 silencing increases oxygen consumption (Fig. 1D), while TRAP1 overexpression reduces it (Fig. 1E), consistent with previous findings obtained in many other cells lines [7, 8, 13, 17, 18], and with the results obtained on complex III basal activity (Fig. 1C). Of note, glucose deprivation decreased respiration rate only in TRAP1 knock-down (shTRAP1) cells, whereas all TRAP1-expressing cells sustain respiration in these conditions (Fig. 1D, E). This finding also recalls the results of complex III activity, and strongly suggests that TRAP1, although reducing complex III basal activity, is important for its function under metabolic stress conditions. Consequently, to assess the contribution of other complexes to the changes in the respiratory profiles obtained by modulating TRAP1 expression, we measured complex I and complex IV activity both upon shRNA-mediated TRAP1 silencing and TRAP1-GFP overexpression in HeLa cells. As a result, the only significant change in activity was observed in complex I, which was decreased upon TRAP1 silencing (Fig. 1F), whereas an increase was observed in complex III activity, thus supporting the key role played by complex III regulation in determining TRAP1-dependent modulation of respiration. Therefore, we measured the viability of HeLa cells following treatment with various concentrations of the complex III inhibitor Antimycin A and the complex I inhibitor Rotenone. Strikingly, we found that shRNA-mediated TRAP1 silencing leads to increased sensitivity to complex III inhibition, and, oppositely, to reduced sensitivity to complex I inhibition (Fig. 1G). These findings strongly support the specificity of TRAP1 role in preserving complex III activity, and its opposite effects on complex I.

To further explore the impact of TRAP1 on complex III, we analyzed expression levels of complex III components by western blot in HeLa cells. We found that TRAP1 silencing leads to increased protein expression of the early assembly factor UQCC1, whose levels are decreased upon TRAP1 overexpression (Fig. 1H). Conversely, expression levels of the catalytic subunit UQCRFS1 (Rieske protein), and the core subunit and TRAP1 partner UQCRC2 were unaffected. As a control, we also looked at the expression of the complex I component NDUFS1, which was also unaffected upon modulation of TRAP1 expression (Fig. 1H). These data suggest that TRAP1 exerts regulation on complex III by direct binding and modulation of activity/assembly, rather than controlling expression/stability of its components.

### TRAP1 binding to UQCRC2 and whole complex III is regulated in different metabolic conditions

**Fig. 2:**
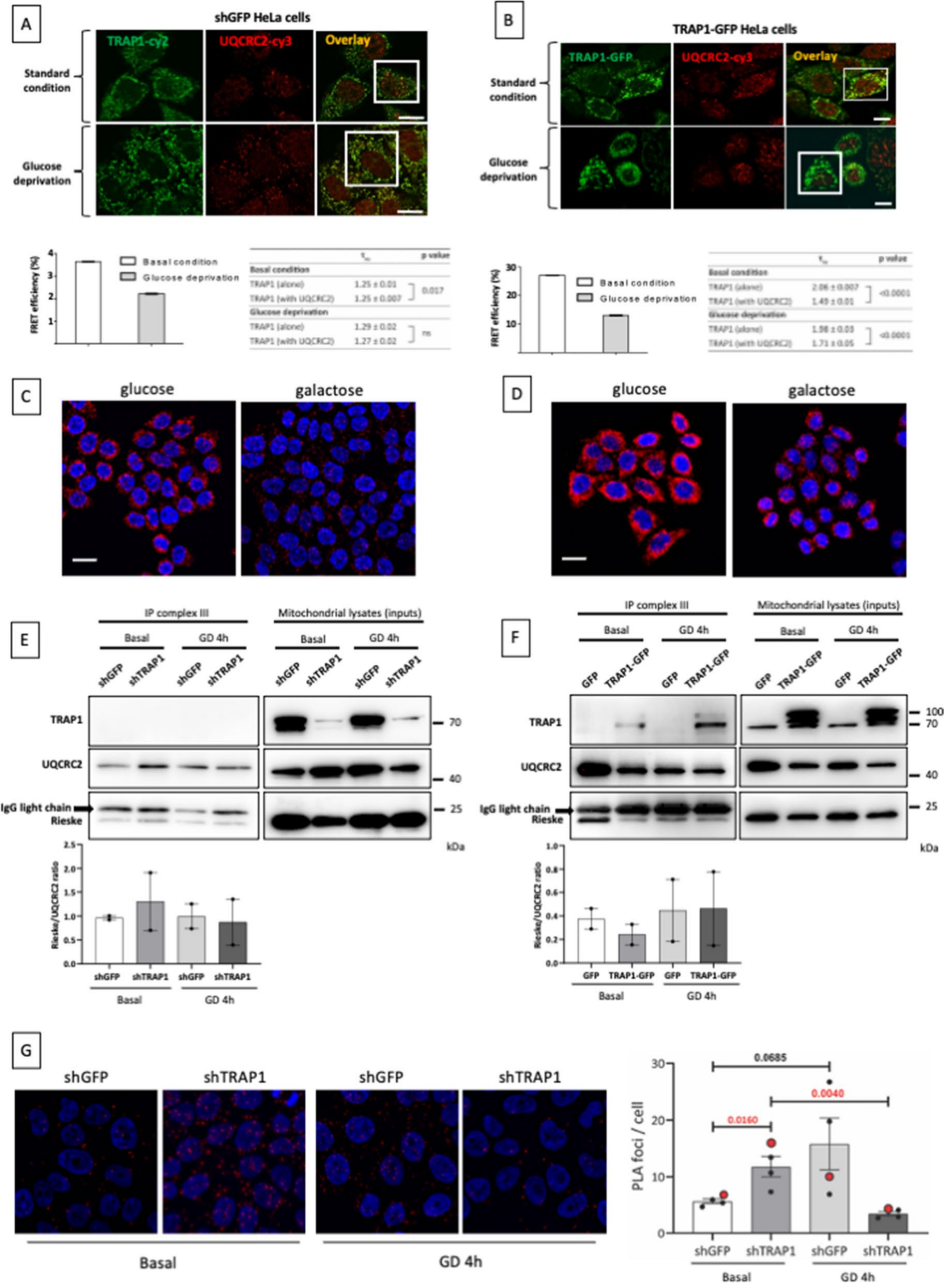
TRAP1 binding to complex III is metabolically regulated. **A**, **B** Fluorescent confocal microscopy analysis of TRAP1 (cy2-donor) and UQCRC2 (cy3-acceptor) (**A**) and of TRAP1-GFP (donor) and UQCRC2 (cy3-acceptor) (**B**) in HeLa cells. Dipole–dipole energy transfer from the fluorescent donor to the fluorescent acceptor allowed calculating FRET efficiency (E_FRET_ %) as described in methods section. The overlay images show the InSet area in which FRET has been analyzed. Scale bars: 10 µm. **τ**_**ns**_ values are expressed as mean ± SEM. Two-tailed *p* values represent the statistical significance based on the Student's t-tests between the **τ**_**ns**_ measured in basal and glucose deprivation conditions (TRAP1 alone versus TRAP1 with UQCRC2). The relative graphs below show the FRET efficiency (percent of control). **C**, **D** Representative image of PLA showing the interaction of UQCRC2 with TRAP1 in HeLa cells (**C**), or in TRAP1-GFP expressing HeLa cells (**D**), in standard culture conditions and following replacement of standard medium with medium containing galactose in the place of glucose (4.5 g/L) for 4 h. Positive signals of interaction are shown as red dots, nuclei are stained with DAPI (blue). Scale bar: 20 µm. **E**, **F** After tetracycline-mediated induction of the expression of TRAP1-directed shRNAs (shTRAP1) or control GFP-directed shRNAs (shGFP) (72 h), or of the expression of the unfused GFP or the TRAP1-GFP fusion protein (24 h), cells were cultured in complete standard medium or low glucose medium (1 g/L) (GD: glucose deprivation) for 4 h and harvested. Intact mitochondria were isolated (see Methods) and lysed for whole complex III isolation. Immunoprecipitates were then subjected to western blot and probed with indicated antibodies. The bar graph in the lower panel shows the ratio between densitometric band intensity of Riske and UQCRC2, expressed as mean ± SEM of two independent experiments. **G** Representative image of PLA showing the interaction of UQCRC2 with Rieske in shGFP/shTRAP1 HeLa cells, in standard culture conditions and following replacement of medium containing low glucose (1 g/L) (GD: glucose deprivation) for 4 h. Positive signals of interaction are shown as red dots, nuclei are stained with DAPI (blue). Scale bar: 10 µm. The bar graph on the right shows the quantification of the PLA foci as mean spot/cell ± SEM (n = 4). Number above bars represent statistical significance obtained by the Student’s t test. The datapoints referred to the representative images shown on the left are evidenced in red

To analyze whether TRAP1 role in the regulation of complex III activity under different metabolic conditions was due to its binding to the complex III core component UQCRC2, we further characterized this interaction in both HeLa control cells and in TRAP1-GFP inducible HeLa cells, in high and low glucose. Using FLIM experiments, we found that TRAP1 and UQCRC2 directly bind to each other; however, this binding is dramatically reduced after culturing cells in low glucose for 4 h, (Fig. 2A, B). Similarly, the replacement of glucose in the medium with an equal amount of galactose, which is known to stimulate respiration [19], dramatically reduced the number of proximity ligation foci between TRAP1 and UQCRC2 (Fig. 2C, D). This result led us to hypothesize that TRAP1 is important for both stability and availability of complex III components, but that functional assembly/activation of the complex requires the removal of TRAP1 from its core. Notably, when we used the TRAP1-GFP overexpression system, the binding between the overexpressed TRAP1-GFP fusion protein and UQCRC2, although reduced to less than a half, was still significant following glucose withdrawal (FRET efficiency: 13%; Fig. 2B); in contrast, such binding is hardly detectable in control cells when glucose is low (Fig. 2A). To test our hypothesis, we performed immunoprecipitation of whole complex III from intact mitochondria isolated from HeLa cells following shRNA-mediated TRAP1 silencing or TRAP1-GFP overexpression in standard and low glucose culture conditions (a representative image of the purity of the mitochondrial preparation is shown in Additional file 1: Fig. S2). Measurements of levels of the catalytic component Rieske, which is added to the pre-complex as the last [20], was evaluated as an indicator of activation. Results showed that, indeed, shTRAP1 cells have slightly higher levels of complex III, detected by both the core component UQCRC2 and the catalytic subunit UQCRFS1 (Rieske protein) (Fig. 2E). However, while the levels of Rieske protein in complex III are decreased in TRAP1 knock-down (shTRAP1) cells following glucose deprivation, these are unchanged in control (shGFP) cells (Fig. 2E), in line with the activity assays. Accordingly, TRAP1-GFP cells have lower levels of complex III, since both UQCRC2 and Rieske are less abundant in the whole complex III IP, but a slight reduction of complex III components following glucose deprivation is present only in control (GFP) cells (Fig. 2F). Surprisingly, we found that, although the direct binding to UQCRC2 decreases upon glucose deprivation (Fig. 2A, B), TRAP1 can still be detected associated to complex III following glucose withdrawal (Fig. 2E, F), suggesting that TRAP1 needs to be moved away from UQCRC2 in order to activate new complex III, but TRAP1 remains bound to the active complex. The ratio between the core subunit UQCRC2 and the catalytic subunit Rieske, as a measure of the proportion between the total complexes and the active ones, well mimics the results obtained by the complex III activity assay (Fig. 2E, F, lower panels). To support these conclusions, we performed PLA between UQCRC2 and Rieske in shGFP/shTRAP1 HeLa cells in the same conditions used for the complex III immunocapture (Fig. 2G). Quantification of PLA foci produced by UQCRC2/Rieske (Fig. 2G, right panel) also well mimics the activity assay, further suggesting a scenario in which TRAP1 already binds UQCRC2 in a pre-complex state, stabilizing and preserving it in the inactive state, to be then displaced upon metabolic demand (such as glucose deprivation) to promote the full assembly of the complex, to the final binding of the catalytic subunit UQCRFS1/Rieske and complex activation.

### TRAP1 regulates metabolic switch depending on nutrient availability

**Fig. 3:**
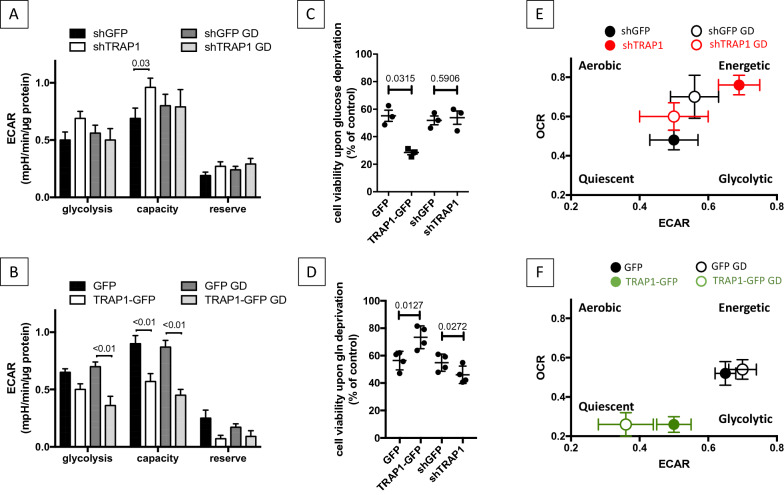
TRAP1 expression is associated to a less energetic metabolism. **A**, **B** Extra cellular acidification rates (ECAR) in HeLa cells following induction of shGFP/shTRAP1 for 72 h (**A**) or GFP/TRAP1-GFP for 48 h (**B**), assayed by the SeaHorse platform as described under “Metabolic analyses” section. Glycolysis, resting ECAR; Glycolytic Capacity, ECAR measured after the addition of oligomycin and FCCP and refers to the maximal glycolytic activity with the OxPhos inhibited; Glycolytic Reserve, difference between ECAR measured in the presence of oligomycin and under resting conditions. The ECAR values were corrected for the residual ECAR measured after the addition of the 2DG glycolytic inhibitor. Before metabolic assay procedures, cells have been cultured in standard culture medium (4.5 g/L glucose) or in low glucose (1 g/L) for 18 h (GD: glucose deprivation). **C**, **D** Viability assays performed in HeLa cells following induction of shGFP/shTRAP1 (72 h) or GFP/TRAP1-GFP (24 h), upon 48 h of complete glucose (**C**) or glutamine deprivation (**D**). The two-tailed *p* value represents the statistical significance based on the Student’s t-test. **E**, **F** Energy map obtained by plotting OCR vs ECAR profiles of shGFP, shTRAP1, GFP and TRAP1-GFP HeLa cells

The data shown above could in part explain some long-standing questions about the still not fully unveiled role of TRAP1 in mitochondrial respiration: in fact, our results show that, depending on glucose availability, TRAP1 expression can correlate with reduced respiration (standard conditions), or with increased respiration (low glucose). Interestingly, the respiratory profiles obtained upon a modulation of TRAP1 expression were accompanied by similar extracellular acidification rate (ECAR) profiles, used as a measure of glycolytic activity (Fig. 3A, B). Glycolytic capacity inversely correlated to TRAP1 expression, with a stronger decrease in shTRAP1 cells in low glucose. Therefore, we analyzed the capacity of TRAP1 overexpressing cells to survive glucose deprivation. As shown in Fig. 3C, the viability of TRAP1-GFP overexpressing cells is significantly reduced after 48 h without glucose. This was anyhow expected, considering the predominant glycolytic profile of these cells and, possibly, that TRAP1 overexpression prevents full displacement of TRAP1-UQCRC2 complex upon glucose withdrawal (Fig. 2B). The observed reduced viability is actually due to the apoptotic cell death, as demonstrated by caspase activity assays (Additional file 1: Fig. S3). In contrast, TRAP1 knock-down cells are more sensitive to deprivation of glutamine, the main energetic source alternative to glucose in cancer cells [21]. Indeed, when cultured for 48 h in the absence of glutamine, the proliferation rate of shTRAP1 cells significantly reduces, compared to shGFP controls (Fig. 3D). In line with these observations, an energy map obtained by plotting OCR versus ECAR upon modulation of TRAP1 levels clearly shows that TRAP1 silencing leads to a more energetic metabolism in standard culture conditions, but also to a more dramatic effect upon glucose deprivation. In contrast, HeLa cells expressing endogenous TRAP1 keep the metabolism unaltered upon glucose withdrawal (Fig. 3E, F). TRAP1 overexpression leads to glucose dependence, possibly because of the persistence of TRAP1-UQCRC2 binding and the subsequent impaired control on the metabolic switch required by reduced glucose availability.

### Complex III protein expression inversely correlates with survival in ovarian cancer

**Fig. 4:**
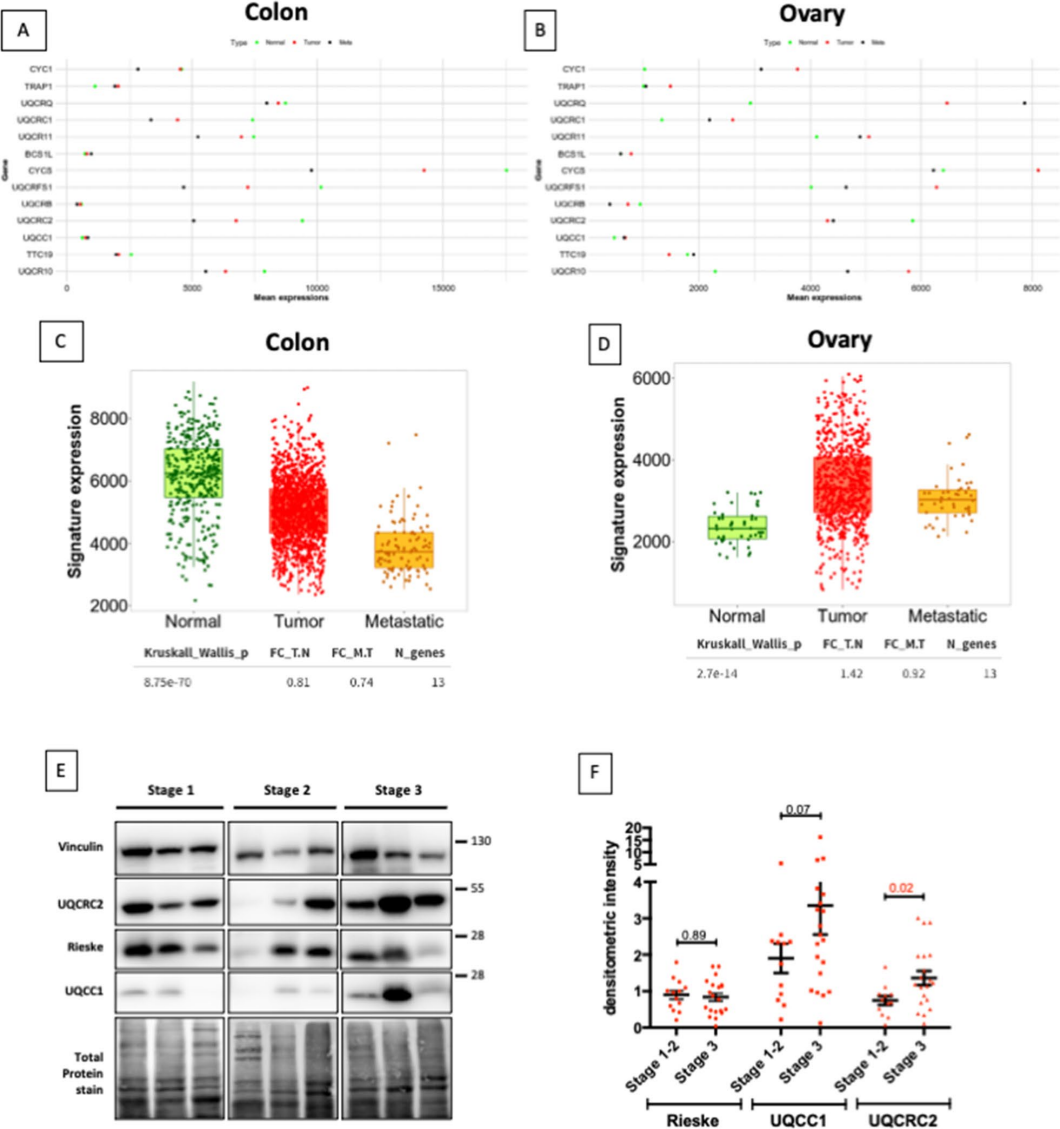
Complex III components are highly expressed in HGSOCs. **A**, **B** Overview of the means of expression of TRAP1, complex III components, and assembly factors in the tumor, normal and metastatic tumor tissues for colon (**A**) and ovary (**B**) using gen chip-based data on TNMplot. **C**, **D** Boxplot graph of gene signature analysis based on the expression of TRAP1, complex III components, and assembly factors (as indicated in panels **A**, **B**) in tumor tissues compared to normal tissues, and metastatic tumors for colon (**C**) and ovary (**D**) based on gene chip data obtained using TNMplot. The statistical significance of differential expression was evaluated by the Kruskal–Wallis test. **E**, **F** Representative blots (**E**) and relative densitometric band quantification (**F**) of total lysates obtained by specimens of stage 1 (n = 7), stage 2 (n = 6), or stage 3 (n = 19) HGSOC patients were analyzed by WB with anti-Rieske, anti UQCC1 and anti-UQCRC2 antibodies, normalized on a reference sample and correlated to clinical parameters of each sample

We have previously shown that TRAP1 inversely correlates with stage and grade and positively correlates with survival in HGSOC [17], and its expression is decreased in metastatic compared to primary tumors [22]. In HGSOC cell models, downmodulation of TRAP1 expression leads to increased respiration rate and induces an OXPHOS-mediated cisplatin resistance, a result that is confirmed by increased BioEnergetic Cellular (BEC) index (i.e. increased oxidative metabolism) of advanced tumors [17]. Conversely, TRAP1 has been well-characterized to facilitate disease progression and induce drug resistance in colorectal cancer [23], where it enhances glycolysis [18]. Based on these data, we decided to explore the TRAP1-complex III axis in these tumors. By using TNM plot [24], we found that expression of several complex III components (data were available for CYCS, CYC1, UQCRB, UQCRC1, UQCRC2, UQCRFS1, UQCRQ, UQCR10, UQCR11) and assembly factors (BCS1L, UQCC1, TTC19) is decreased in colon tumor tissues compared to the normal tissue, and is further decreased in metastatic tumors compared to the primary ones (Fig. 4A–C). On the contrary, ovarian tumors show overall increased expression of complex III components and assembly factors, whereas not significant alteration of their expression is observed in metastatic tissues compared to the primary tumors (Fig. 4B–D). A similar scenario is also observed when complex I, II and IV components and assembly factors are analyzed (Additional file 1: Fig. S4). These data support the idea that OC tend to rely more on oxidative phosphorylation compared to other tumors with a classical Warburg phenotype—among those, colorectal cancer [25]—and that TRAP1 is accordingly co-regulated, being reduced in oxidative tumors (as expected, given its inhibitory role on basal respiration) and upregulated in the glycolytic ones. We then analyzed the expression of some complex III components and assembly factors by western blot in a set of tissue samples obtained from HGSOC biopsies at various stages, that had been previously characterized for their metabolic profile [17]. We found that stage 3 tumors display higher (though not statistically significant) expression of UQCC1, and significantly higher expression of UQCRC2 compared to stage 1–2 tumors, whereas Rieske expression levels were comparable between the two groups (Fig. 4E, F).

**Fig. 5:**
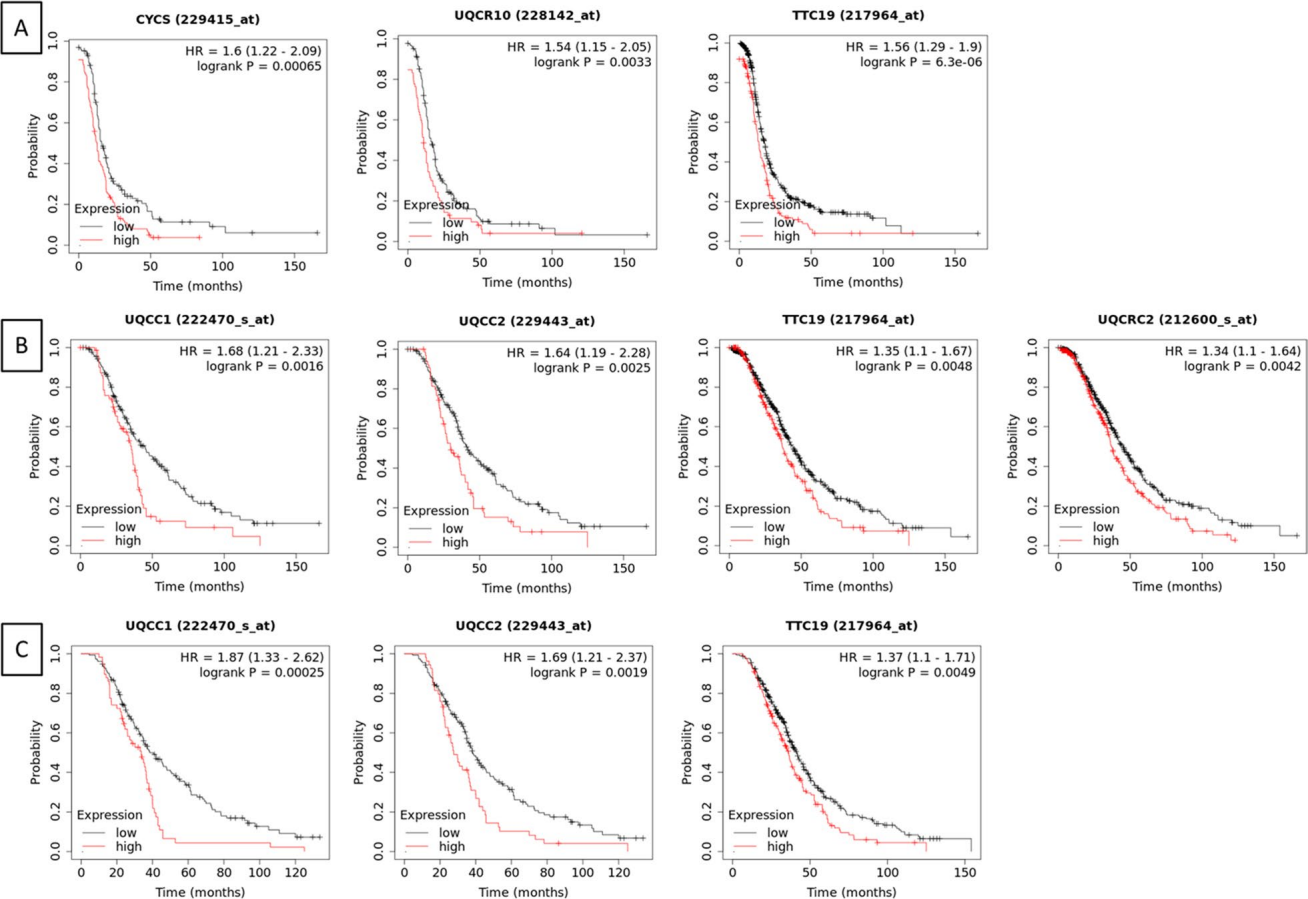
Expression of several complex III components or assembly factors inversely correlates with survival in late-stage HGSOCs. Kaplan–Meier estimates of survival obtained on Kaplan Meier plotter analyzing progression-free survival (**A**), overall survival (**B**) and post-progression survival (**C**) in HGSOC, restricting analysis to stage 3–4 patients treated with platinum-based chemotherapy

Starting from this preliminary observation, we investigated the impact of complex III components on the outcome of stage 3 HGSOC. By using Kaplan Meier plotter [26], we found that high expression of CYCS, UQCR10 and TTC19 significantly correlates with a worse progression-free survival (Fig. 5A), high expression of UQCC1, UQCRC2, TTC19 and UQCC2 significantly correlates with worse overall survival (Fig. 5B), and high expression of UQCC1, UQCC2 and TTC19 correlates with worse post-progression survival (Fig. 5C). Notably, the assembly factor TTC19 inversely correlates with all the three survival parameters.

### TRAP1 and complex III have opposite effects on platinum sensitivity in HGSOC

**Fig. 6:**
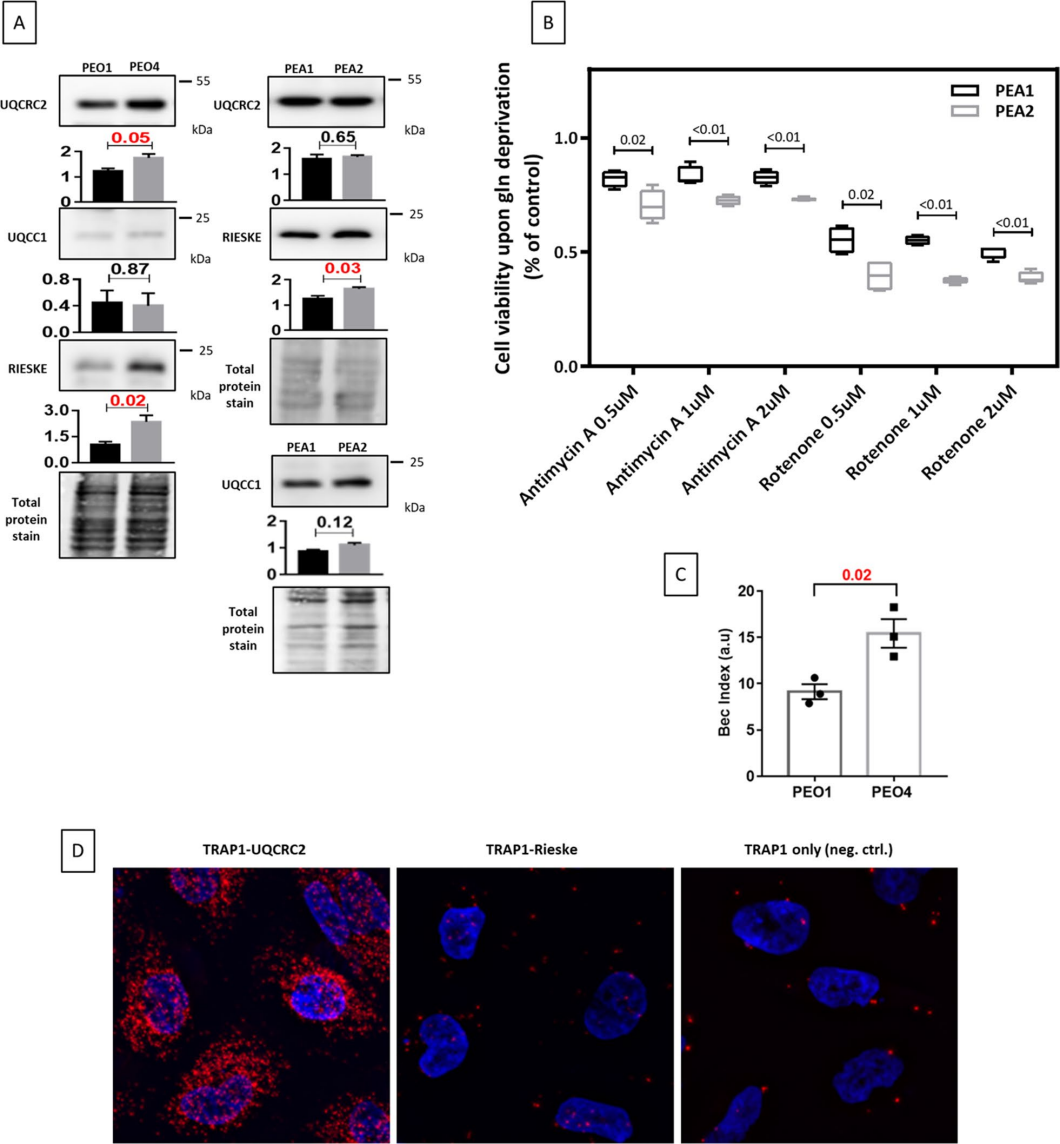
Platinum-resistant HGSOC cells display high complex III expression/activity. **A** Total lysates obtained from platinum-sensitive PEA1 and matched platinum-resistant PEA2 cells or from platinum-sensitive PEO1 and matched platinum-resistant PEO4 cells were separated by SDS-PAGE and immunoblotted with the indicated antibodies. Images are representative of four independent experiments. Bar graphs below each image represent densitometric quantification of bands, expressed as mean ± SEM (n = 4). Numbers above bars indicate the statistical significance (*p* value) obtained by the Student’s t-test (significant *p* values are highlighted in red). **B** Viability assays performed in cisplatin-sensitive PEA1 and cisplatin-resistant PEA2 cells following treatment with Antimycin A and Rotenone at increasing concentrations for 48 h. Data are expressed as mean ± SEM of four independent experiment, with technical triplicates each. Numbers indicate the statistical significance (*p* value) obtained by the Student’s t-test (significant results are highlighted in red). **C** BEC index analysis on PEO1 and PEO4 cells. Cells were harvested and total protein lysates were immunoblotted with anti-βF1ATPase, anti-HSP60 and anti-GAPDH antibodies. Immunoreactive bands were quantified by using ImageJ and BEC index was calculated by the formula F1ATPase/HSP60/ GAPDH (see Material and Methods section for details). Data are expressed as mean ± S.E.M. from three independent experiments. Numbers above bars indicate the statistical significance (*p* value), based on the Student’s t-test. **D** Representative image of PLA showing the interaction of TRAP1 with UQCRC2 or Rieske in PEA1 cells. Positive signals of interaction are shown as red dots, nuclei are stained with DAPI (blue). Negative control has been obtained by hybridizing cells with TRAP1 antibody only. Scale bar: 10 µm

These analyses prompted us to evaluate the relevance of complex III in HGSOC cells. To this aim, we took advantage of two different couples of matched pair of cisplatin sensitive/resistant isogenic cell lines obtained from the same patient before and after chemotherapy (PEA1/PEA2, PEO1/PEO4), that well recapitulate the characteristics of clinically-acquired platinum resistance [27]. We firstly characterized the expression of complex III components and assembly factors by western blot in the matched cell lines, showing that Rieske expression is increased in the both the resistant PEA2 and PEO4 compared to their sensitive counterparts PEA1 and PEO1, and that UQCRC2 is increased in PEO4 compared to PEO1, while UQCC1 expression is unchanged (Fig. 6A). In order to verify that this finding correlates with a higher activity of the complex and higher dependence of chemoresistant cells on complex III and, more broadly, on respiration, we treated the cell lines with Antimycin A or Rotenone, well-known complex III and complex I inhibitors, respectively, and found that cisplatin-resistant PEA2 cells, that have been previously characterized for their increased oxidative metabolism-induced/dependent chemoresistance [17], and show several links between altered metabolism and chemoresistance [28, 29], are actually more sensitive to both compounds, in terms of cell viability (Fig. 6B). Of note, we confirmed that, similarly to the PEA1/PEA2 couple, PEO4 cells display higher oxidative metabolism than PEO1 since the BEC index is significantly higher in the chemoresistant cells (Fig. 6C) [29]. Considering that we have previously demonstrated that TRAP1 plays an important role in the acquisition of OC chemoresistance through the regulation of oxidative phosphorylation [17], and it is negatively selected by cisplatin treatment, we confirmed TRAP1-UQCRC2 selective binding in this cell system by PLA. In keeping with the results obtained in HeLa cells, TRAP1-UQCRC2 indeed produced strong PLA signals, whereas TRAP1-Rieske PLA was not different from the negative control (Fig. 6D).

**Fig. 7:**
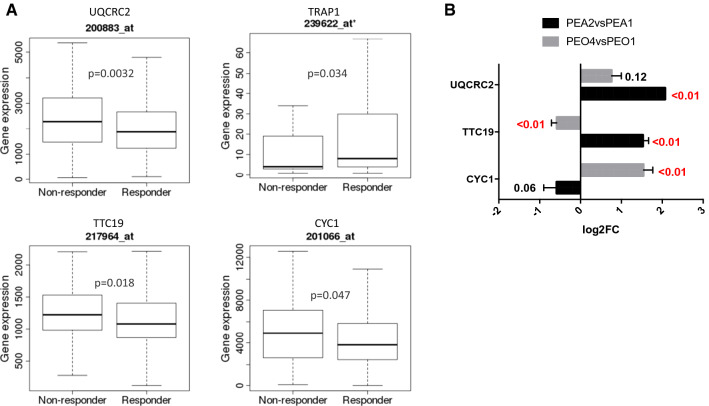
High complex III expression is associated to resistance to platinum-based therapy in HGSOC. **A** Expression levels of UQCRC2, TRAP1, TTC19 and CYC1 in HGSOC patients at stage 3, grouped for their pathological response to platinum-based therapy (complete response vs residual disease after completing the therapy). Statistical significance of differences in gene expression levels in responders (n = 366) and non-responders (n = 90) was evaluated by the Mann–Whitney test. **B** Real-time RT-PCR analysis of indicated genes in platinum-resistant PEA2 and PEO4 compared to their matched-sensitive counterparts PEA1 and PEO1. Data are expressed as mean ± S.E.M. of − ΔΔCt (log2FC) from four independent experiments with technical triplicates each. Numbers indicate the statistical significance (*p* value), based on Student’s t-test (significant values highlighted in red)

Supported by these in vitro observations, we searched for the correlation between gene expression and response to therapy using transcriptome-level data on ROC plotter [30]. These analyses allowed us to observe that complex III core component UQCRC2, the catalytic subunit CYC1, and the assembly factor TTC19 are expressed at a significantly higher level (FC = 1.2, FC = 1.2, FC = 1.1, respectively) in patients who do not respond to platinum-based therapy (non-responders), where TRAP1 is expressed at a significantly lower level (FC = 1.8) (Fig. 7A), in keeping with our previous findings [17, 22]. Accordingly, qPCR analysis of the same genes in our matched cell lines confirmed significantly higher mRNA levels of UQCRC2 and TTC19 in PEA2, while CYC1 is higher in PEO4 than in PEO1 (Fig. 7B).

## Discussion

TRAP1 is the main mitochondrial member of the HSP90 protein family, where it interacts with respiratory complexes [4, 13]. It is also partly localized on the endoplasmic reticulum, where it is involved in the regulation of protein synthesis through the binding to components of the translational machinery [31]. Interest in TRAP1 has considerably grown in recent decades due to its contextual effects in different tumor types: it is highly expressed in several cancers and correlated with drug resistance, but is downregulated in specific tumors with predominant oxidative metabolism [6].

Here we have further characterized the binding of TRAP1 to respiratory complexes and demonstrated for the first time its interaction with complex III, increasing our knowledge of the impact of TRAP1 regulation of the respiratory chain activity [4]. Remarkably, we show that TRAP1 is involved in the regulation of mitochondrial respiration through a direct binding to UQCRC2, a specific complex III core component, affecting complex assembly. Indeed, TRAP1-UQCRC2 binding is regulated by the metabolic demand, with detachment of the two proteins from each other upon glucose deprivation. A possible explanation for this regulation is that TRAP1 binding to UQCRC2 only occurs in the pre-complex, to preserve partially assembled complex III available for spare compensatory respiration upon metabolic stress, although further studies are required to identify the specific assembly step and molecular mechanisms involved in this regulation. Accordingly, it has been previously shown that TRAP1 silencing increases respiration, but reduces spare respiratory capacity, bringing basal respiration close to maximal [8], and that it is important for succinate dehydrogenase stability and activity when limiting glucose induces compensatory mitochondrial metabolism [9]. Upstream signaling potentially responsible for this regulation also needs further research, but it has been already demonstrated that TRAP1 can be subject to diverse post-translational modification modulating its activity [32, 33], one of those being mediated by the PINK1 kinase [34], whose loss also cause dysregulation of mitochondrial respiration [35].

The biogenesis of complex III is initiated by the mitochondrial ribosome synthesis of cytochrome b and its co-translational translocation to the mitochondrial inner membrane, which is assisted by UQCC1 and UQCC2 [36]. Notably, we found that UQCC1 is markedly increased upon TRAP1 silencing, and decreased upon TRAP1 overexpression. However, glucose deprivation induces a dramatic decrease in global translation [37], therefore de novo synthesis of complex III component is unavailable for active complex assembly under prolonged stress conditions, and may require ad hoc stress-responsive mechanisms. In this view, our study suggests a new scenario in which TRAP1 binds UQCRC2 in a pre-complex, stabilizing and preserving it in the inactive state; then, a displacement between the two proteins occurs upon metabolic demand (such as glucose deprivation or its replacement with galactose) to promote full complex assembly and activation, as finally evidenced by the recruitment of the catalytic subunit UQCRFS1/Rieske.

Several lines of evidence suggest increasing correlations between metabolic rearrangements and cancer, with TRAP1 playing an important (but only partially understood) role at a crossroad between glycolytic and OXPHOS metabolic regulation in a tumor-specific way. This study provides evidence for further molecular mechanisms involved in TRAP1-dependent regulation of cancer cell metabolism, and points out complex III components and activity as a potential novel target for metabolic therapy, especially in ovarian cancer. In basal conditions, all the structural subunits are added to the complex until only UQCRFS1 and UQCR11 are missing, in an already dimeric structure named pre-cIII_2_ [38]. The assembly of ultimately active complex III then requires the insertion of the Fe-S Rieske protein (UQCRFS1) between the two major core components UQCRC1 and UQCRC2, which is assisted by LYRM7 [39], with subsequent cleavage and partial removal of the UQCRFS1 mitochondrial targeting sequence, a process in which TTC19 plays a key role [40]. Interestingly, we have found that high expression of TTC19 assembly factor significantly correlates with lower survival of advanced stage-HGSOC patients following platin-based therapy, and that its expression is significantly higher in non-responder patients. UQCRC2, which is potentially responsible for the cleavage of the UQCRFS1 mitochondrial targeting sequence [40], also negatively correlates with overall survival and response to therapy, whereas TRAP1 is decreased in non-responders to platinum-based therapy. This points towards a homeostatic role for TRAP1 in predominantly glycolytic metabolisms, in which TRAP1-UQCRC2 interaction preserves core components for the de novo assembly of active complex III in conditions of metabolic stress, as a strategy to activate compensatory respiration; on the opposite, TRAP1 expression is counter-selected in mostly respiratory contexts, in which higher basal respiration is preferred. Relevant to this “metabolic stress” model, the interchange of catalytically active subunits, that can be more easily oxidatively damaged, by replacement of old ones with newly imported ones on a pre-assembled complex, could ensure complex functionality, as already demonstrated for complex I [41]. Given its specific role in the final addition of the Rieske protein on the already dimeric pre-complex III (pre-cIII_2_) [40], the importance of the assembly factor TTC19 in tumor cells highly relying on complex III function, as suggested by our analyses, can be justified; whereas TRAP1 could be important in glycolytic tumor cells for “salvage” respiration when glucose is unavailable. According to this model, overexpression of TRAP1 and consequent (partial) persistence of TRAP1-UQCRC2 binding upon glucose withdrawal could lead to glucose dependence. In general, we have observed that high expression of TRAP1 correlates with a less active metabolic profile; conversely, reduced TRAP1 expression yields a more ‘energetic’ metabolism, with enhanced oxidative phosphorylation, but a reduced response to nutrient limitation. As a result, TRAP1 expression directly correlates with sensitivity to glucose withdrawal, whereas an inverse correlation occurs to glutamine deprivation.

## Conclusions

It has been recently proposed that complex III is central for mitochondrial respiratory chain maturation, suggesting a cooperative-assembly model in which super-complexes are formed thanks to the structural and functional platform provided by complex III, and assigning to this specific complex a central role for the completion of overall mitochondrial respiratory chain biogenesis [42]. Complex III is also selectively targeted by the crucial regulation operated on electron transport chain maturation and function by the novel small peptide BRAWNIN [43]. Furthermore, beyond its mechanistic function in the electron transport chain, complex III shows peculiar and important functions in cellular signaling: loss of complex III in T_reg_ compromise their suppressive function and immune regulation, by increasing DNA methylation through the accumulation of metabolites 2-hydroxyglutarate and succinate that inhibit the ten-eleven translocation family of DNA demethylases, proportionally more than complex I and II [44]. This suggests that modulation of complex III can induce profound changes in gene expression, and therefore correlation between complex III activity/expression in advanced tumor stages and drug resistance, especially in highly oxidative tumors, deserves further attention.

The results reported herein partially solve present controversies on TRAP1 functions in the regulation of energetic metabolism, and provide novel elements to shed light on new molecular mechanisms underlying this control, as well as novel targets to be explored for ovarian cancer therapy.

## Methods

### Cell cultures

Human HCT116 colon carcinoma cells and human cervical carcinoma HeLa cells were purchased from American Type Culture Collection (ATCC) and cultured in McCoy's 5A medium (HCT116) and DMEM (HeLa). Both culturing mediums contain 10% fetal bovine serum, 1.5 mmol/L glutamine. The authenticity of the cell lines was verified by STR profiling, in accordance with ATCC product description. HeLa Flp In TRex (FITR) cell line were kindly provided by Dr. Matthias Gromeier (Duke University Medical Center, Durham, USA). Generation of the HeLa Flp In TRex stable cell lines expressing the eGFP-fusion proteins or the short hairpin RNA, was performed as described in the manufacturer’s protocol (Flp In TRex, Invitrogen). HeLa Flp In TRex cells were cultured in DMEM supplemented with 10% fetal bovine serum, 1.5 mmol/L glutamine, and appropriate selective antibiotics. Addition of tetracycline induces proteins/shRNAs expression.

The paired HGSOC cell lines PEA1/PEA2, PEO1/PEO4 have been described elsewhere [27], and were maintained in RPMI 1640 media with 10% fetal bovine serum, glutamine and Normocin (Invivogen), at 37 °C, 5% CO_2_.

### Plasmid generation and transfection procedures

For TRAP1-eGFP plasmids generation, HeLa cDNA library and eGFP plasmid were used as templates for fusion PCR. Resulting chimeric cDNAs were cloned into pCDNA5/FRT/TO. pFRT-U6tetO is a kind gift from prof. John J Rossi. Inducible shRNA generated as described in [45] (using BglII/KpnI as restriction sites). Short hairpin sequences used are: GFP=agatctGCACAAGCTGGAGTACAACTACCTGACCCATAGTTGTACTCCAGCTTGTGCTTTTTggtacc; TRAP1=agatctGCCCGGTCCCTGTACTCAGAAACCTGACCCATTTCTGAGTACAGGGACCGGGCTTTTTggtacc.

For Transient transfection of DNA plasmids was performed with the Polyfect Transfection Reagent (Qiagen—301105) according to the manufacturer's protocol. TRAP1 transient silencing was performed with siRNAs purchased from Qiagen (cat. no. SI00115150). For control experiments, cells were transfected with a similar amount of scrambled siRNA (Qiagen; cat. no. SI03650318). Transient transfections of siRNAs were performed using HiPerFect Transfection Reagent (Qiagen—301704) according to the manufacturer's protocol. The flag-UQCRC2 construct was generated by cloning the UQCRC2 gene between EcoRI and BamHI restriction sites in p3xFlag-CMV-7.1 expression vector. The UQCRC2 gene was amplified by RT-PCR from total HeLa RNAs with the following primers containing the above mentioned restriction sites: forward: 5′-ATTAGAATTCAATGAAGCTACTAACCAGAGCCGG-3′; reverse: 5′-ATTAGGATCCTTACAACTCATCAACAAAAGGTGTATGTCCC-3′.

### Western blot and immunoprecipitation

Equal amounts of protein from cell lysates were subjected to SDS-PAGE and transferred to a PVDF membrane (Millipore). Protein immunoprecipitations were carried out as previously described [46]. GFP-fusion proteins were immunoprecipitated with GFP-trap magnetic agarose beads (GFP-trap_MA Chromotek) according to manufacturer’s instructions. Flag-UQCRC2 was immunoprecipitated with anti-flag M2 magnetic beads (Sigma, M8823) according to manufacturer’s instructions. Immunoprecipitation of whole complex III was performed by using Anti-Complex III Immunocapture antibody (Abcam, ab109862) from intact isolated HeLa cell mitochondria by using 1% digitonin as detergent (see “Cell fractionation” section below) according to manufacturer’s instructions. Immunocomplexes were then isolated using Protein G Dynabeads (Thermo Fisher Scientific, 10003D). Where indicated, protein levels were quantified by densitometric analysis using the software ImageJ [47]. The following antibodies were used for WB, microscopy observations and immunoprecipitation: anti-TRAP1 (Santa Cruz Biotechnology, sc-13557), anti-β-ACTIN (Santa Cruz Biotechnology, sc-69879), anti-HSP60 (Santa Cruz Biotechnology, sc-1052), anti-phospho-AMPKα (Thr172) (Cell Signaling Technology, #2531), anti-BCS1L (Santa Cruz Biotechnology, sc-134280), anti-Rieske (Santa Cruz Biotechnology, sc-271609), anti-UQCC1 (Bethyl n. A305-430A), anti-UQCRC2 (Genetex, GTX114873), anti-GFP (Santa Cruz Biotechnology, sc-81045), anti-ATP5B (Santa Cruz Biotechnology, sc-55597), anti-GAPDH (Santa Cruz Biotechnology, sc-69778), anti-NDUFS1 (Santa Cruz Biotechnology, sc-99232), anti-PHB2 (Santa Cruz Biotechnology, sc-133094), anti-Flag (Sigma, SAB4301135). Images were acquired using the ChemiDoc MP system (Bio-Rad). Where indicated, loding of proteins into gels were quantified using The No-Stain™ Protein Labeling Reagent (Thermo Fisher Scientific).

### Duolink in situ proximity ligation assay

Duolink in situ proximity ligation assay (Sigma-Aldrich—DUO92101) was performed according to the manufacturer’s instructions. Briefly, cells were seeded on coverslips, fixed, permeabilized and hybridized with primary antibodies. After one day, cells were hybridized with secondary antibodies conjugated with the PLA probes (PLUS and MINUS), and then subjected to ligation and rolling circle amplification using fluorescently labelled oligonucleotides. Cells were washed and mounted on slides using a mounting media with DAPI to detect nuclei and signal was detected by confocal microscopy analysis. For proximity ligation assays, the following antibodies were used: anti-TRAP1 (sc-13557), anti-TRAP1 (Genetex, GTX102017), anti-UQCRC2 (Genetex, GTX114873) and anti-Rieske (sc-271609).

### Cell fractionation

Mitochondria were purified by using the Qproteome Mitochondria Isolation kit (Qiagen—37612) according to the manufacturer’s protocol.

### Complex III activity assay

Intact mitochondria were isolated from HeLa cells by using the Qproteome Mitochondria isolation kit (Qiagen, Cat. No 37612) according to the manufacturer’s manual. The complex III activity was measured by using the Mitochondria Complex III Activity Assay Kit (Biovision, Cat. No. K520). In brief, we added cytochrome c in samples containing 3–6 µg of isolated mitochondria and recorded the absorbance of the reduced cytochrome c at 550 nm, at 30-s intervals for 10 min at RT. Antimycin A inhibitor and DMSO were used in negative and background control samples, respectively. Complex III specific activity was calculated by applying the following formula:

$${\text{Net}}\;{\text{complex}}\;{\text{III}}\;{\text{specific}}\;{\text{activity}} = {\text{Complex}}\;{\text{III}}\;{\text{specific}}\;{\text{activity}}\;{\text{w}}/{\text{o}}\;{\text{Antimycin}}\;{\text{A}}{-}{\text{Complex}}\;{\text{III}}\;{\text{specific}}\;{\text{activity}}\;{\text{with}}\;{\text{Antimycin}}\;{\text{A}}.$$

Complex III specific activity was calculated by applying the following equation:

$${\text{Complex}}\;{\text{III}}\;{\text{specific}}\;{\text{activity}} = \Delta {\text{C}}/\left( {\Delta {\text{t}}*{\text{p}}} \right)*{\text{D}}$$

where ∆C = change in reduced cytochrome c concentration during the ∆t; ∆t = t2 − t1 (min); p = mitochondria protein sample (µg); D = dilution factor.

### Complex I and complex IV activity assay

Hela cells (shGFP/shTRAP1 and GFP/TRAP1-GFP) were collected, resuspended in 0.32 M sucrose, 40 mM KCl, 20 mM Tris–HCl, 2 mM EDTA pH 7.2 at 5–10 10^6^ cell/mL and subjected to ultrasound treatment on ice by Sonics Vibracell ultrasonic cell disruptor following manufacturer instructions. The obtained homogenates were diluted 1:5 for complex I activity and 1:8 for complex IV activity in 0.4 mL of the assay buffer constituted by 10 mM Tris–HCl, 1 mg/mL BSA, pH 7.4. Measurements were carried out spectrophotometrically as in [48]. Briefly, for complex I activity, 50 μM of NADH was added to the cell suspension in the absence or in the presence of 2 μM rotenone, and the absorbance decreases followed at 340 nm and converted in nmoles of NADH oxidation by using an ε = 6.22 mM cm^−1^; the specific complex I activity was attained by correction for the rotenone insensitive activity. For complex IV activity, 20 μM ferrocytochome c (reduced form) was added to the cell suspension in the absence or in the presence of 3 mM KCN, and the absorbance decreases followed at 550 nm and converted in nmoles of ferrocytochrome c oxidation by using an ε = 19.1 mM cm^−1^; the specific complex IV activity was attained by correction for the KCN insensitive activity. The reduced form of cytochrome c was attained by adding a few grains of sodium dithionite to 2.5 mM cytochrome c and dialyzed overnight. For citrate synthase activity the homogenates were diluted 1:8 in 0.4 mL of 100 mM Tris–HCl pH 8.0 supplemented with 0.3 mM acetyl-CoA, 0.1 mM 5,5′-Dithiobis(2-nitrobenzoic acid) (DTNB), 0.2% Triton X-100 in the presence or absence of 0.5 mM oxalacetate (OAA) and the absorbance increase followed at 412 nm and converted in nmoles of TNB formation by using an ε = 13.6 mM cm^−1^ (that is a measure of the citrate synthase activity according to: OAA + Acetyl-CoA + DTNB → citrate + TNB + CoA-S-TNB); the specific citrate synthase activity was attained by correction for the absorbance increase in the absence of OAA. All the activities were normalized to the mg of proteins, assayed by the Bradford method, and the activities of complexes I and IV normalized to that of the citrate synthase.

### Metabolic analyses

The metabolic profile has been evaluated in Hela cells after 72 h (shGFP/shTRAP1) or 48 h (GFP/TRAP1-GFP) of Tet induction. Real-time measurements of OCR and ECAR were made using an XF-96 Extracellular Flux Analyzer (Seahorse Bioscience, North Billerica, MA, USA). Cells were plated in XF-96 plates (Seahorse Bioscience) at a concentration of 15,000 cells per well and cultured for 12 h in DMEM medium supplemented with 5% FBS. For OCR analysis, after replacing the growth medium with 180 μL of bicarbonate-free DMEM supplemented with 10 mM glucose 2 mM l-glutamine and 1 mM sodium pyruvate pre-warmed at 37 °C, cells were preincubated for 45 min before starting the assay procedure. After measuring basal respiration, oligomycin (1 μM), carbonyl cyanide m-chlorophenylhydrazone (1 μM), and rotenone + antimycin A (1 μM + 1 μM) were injected into each well sequentially to assess respectively coupling of the respiratory chain, maximal and non-mitochondrial oxygen consumption. Glycolytic flux (basal glycolysis, glycolytic capacity, and glycolytic reserve) was analyzed by the sequential addition of 10 mM glucose, 1 μM oligomycin, and 100 mM 2-deoxyglucose. Experiments with the Seahorse system have been performed with the following assay conditions: 3-min mixture; 3-min wait; and 3-min measurement; metabolic parameters were then calculated. The values were normalized to protein content in each well, determined with BCA assay. Data are expressed as mean ± S.E.M.

The BioEnergetic Cellular (BEC) index of the cell lines were calculated, based on densitometric quantification of digitally acquired western blots images as described above, by dividing the ratio of βF1ATPase to HSP60 with the GAPDH value, as previously described [49].

### Förster resonance energy transfer (FRET) assay by fluorescence lifetime imaging (FLIM)

In FRET experiments, the TRAP1-GFP fusion protein or a cy2 conjugated to a secondary antibody were used as donor, while cy3 conjugated to a secondary antibody was used as acceptor. The cells were fixed in 2% paraformaldehyde (PFA), mounted on a slide and analyzed using a TCS SMD FLIM Leica SP5 microscope (Leica, Wetzlar, Germany) equipped with a 63X/1.4 NA objective, to measure the efficiency of FRET. FRET efficiency (E_FRET_) varies as the sixth power of the distance (r) between the two molecules according to the following formula: E_FRET_ = 1/[(1 + r/R_0_)^6^], where R_0_ is the distance corresponding to E_FRET_ = 50%, which can be calculated for any pair of fluorescent molecules. For distances less than R_0_, FRET efficiency is close to maximal, because of the 1/r^6^ dependence, whereas for distances greater than R_0_ the efficiency is close to zero. In particular, the FRET efficiency by FLIM was calculated with the following formula: E_FRET_: 1 − t_DA_/ t_D_, where t_DA_ is the donor lifetime in presence of the acceptor, while t_D_ is the lifetime of the donor alone.

### Cell treatments and apoptosis and viability assays

In glucose deprivation experiments, cells were plated in monolayer in complete medium. After seeding, medium was replaced with low glucose (1 g/L) medium or, for viability and apoptosis assays only, with DMEM medium without glucose (Sigma), or with medium containing the indicated concentrations of Antimycin (Sigma, A8674) or Rotenone (Sigma, R8875). Cell viability was measured by MTT assay by using the In vitro toxicology assay kit (Sigma, product code TOX1-1KT), following the manufacturer's instructions. Apoptosis was measured using the Caspase-Glo 3/7 assay (Promega, Milano, Italy, product code G8090) and was performed according to the manufacturer's instructions.

### Bioinformatic analyses

Differential gene expression analysis in tumor, normal and metastatic tissues was performed by using TNMplot [24]. Survival estimates were obtained by Kaplan–Meier plotter [26] for ovarian cancer, with “auto-select best cutoff”, histology “serous”, grade 3, stage 3 + 4, therapy containing platin, and excluding biased arrays. The links between gene expression profile obtained by transcriptomic data and response to therapy have been searched by using ROC plotter [30] for ovarian cancer, selecting “serous” for histology, grade 3, stage 3, platin-based therapy, and looking for pathological response, with “no outliers”. Only the genes with a *p* < 0.05 based on the Mann–Whitney test were selected.

### RNA extraction and real-time reverse transcriptase-polymerase chain reaction (RT-PCR)

Total RNA extraction procedures were performed by using TRI Reagent (Merck Life Science S.r.l., Milano, Italy; product code T9424), following the manufacturer’s instruction. For first-strand synthesis of cDNA, 1 μg of RNA was used in a 20-μL reaction mixture by using a SensiFast cDNA synthesis kit (Bioline, London, UK). For real-time PCR analysis, 0.4 μL of cDNA sample was amplified by using the SensiFast Syber (Bioline, London, UK) in an iCycler iQ Real-Time Detection System (Bio-Rad Laboratories GmbH, Segrate, Italy). The reaction conditions were 95 °C for 2 min followed by 40 cycles of 5 s at 95 °C and 30 s at 60 °C. PPIA was chosen as the internal control. The following primers were used for PCR analysis:

**Table:** 

PPIA:	Forward: 5′-CTGCACTGCCAAGACTGA-3′;	Reverse: 5′-GCCATTCCTGGACCCAAA-3′
CYC1:	Forward: 5′-TACGGACACCTCAGGCAGTG-3′;	Reverse: 5′-CACGGTGAGACCACGGATAG-3′
TTC19:	Forward: 5′-TTTGCATGACGCTCTTCGTC-3′;	Reverse: 5′-TGCATTGTCCTCCTGCTTCAT-3′
UQCRC2:	Forward: 5′-CTTACCGGAATGCCTTGGCT-3′;	Reverse: 5′-GATAAACCAAGCCCACCCCT-3′

## Supplementary Information

**Additional file 1. Supplementary Figures**

**Additional file 2. Original blots**

## Data Availability

All data generated or analysed during this study are included in this published article and its additional information file. Original, uncropped and unadjusted blots are reported in Additional File 2.

## Abbreviations

BEC index: BioEnergetic Cellular Index
HGSOC: High Grade Serous Ovarian Cancer
shRNA: Short hairpin RNA
OXPHOS: Oxidative phosphorylation
