# Supplementary material for: Regulation of mitochondrial complex III activity and assembly by TRAP1 in cancer cells
Source: Cancer Cell Int. 2022 Dec 12;22:402. doi: 10.1186/s12935-022-02788-4 (PMC9743594; doi:10.1186/s12935-022-02788-4)
Supplement: Supplementary file 1 — Additional file 1. Supplementary Figures [file 12935_2022_2788_MOESM1_ESM.pdf]

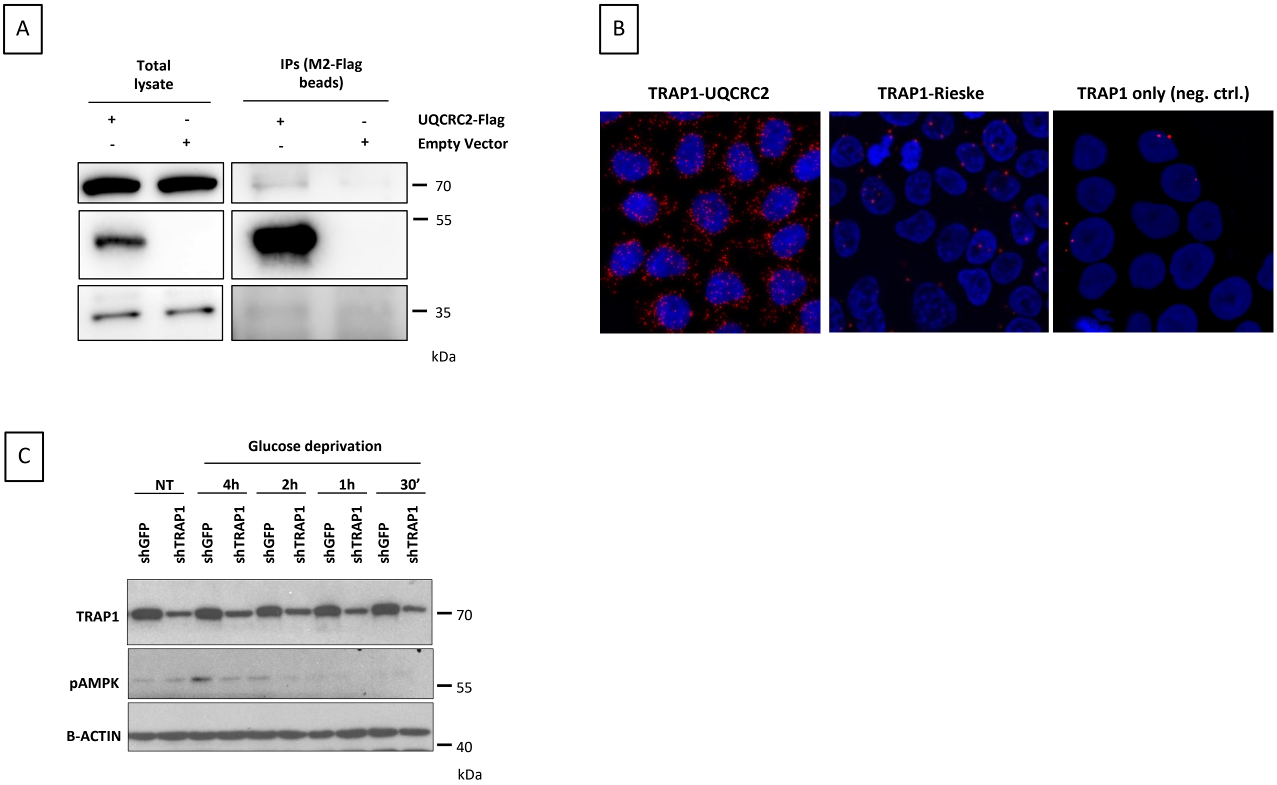

Supplementary Figure 1: TRAP1 specifically binds the complex III core component UQCRC2. (**A**) Co-immunoprecipitation of a flagged UQCRC2 with TRAP1 performed in HeLa cells: total lysates were incubated with flag antibody-conjugated beads and the resulting samples were immunoblotted with indicated antibodies. (**B**) Representative image of PLA showing the interaction of TRAP1 with UQCRC2 or Rieske in HeLa cells. Positive signals of interaction are shown as red dots, nuclei are stained with DAPI (blue). Negative control has been obtained by hybridizing cells with TRAP1 antibody only. Scale bar: 10 µm**. (C)** WB analysis of HeLa cell extracts following shGFP or shTRAP1 (72 hrs) induction. Total lysates were immunoblotted with indicated antibodies.

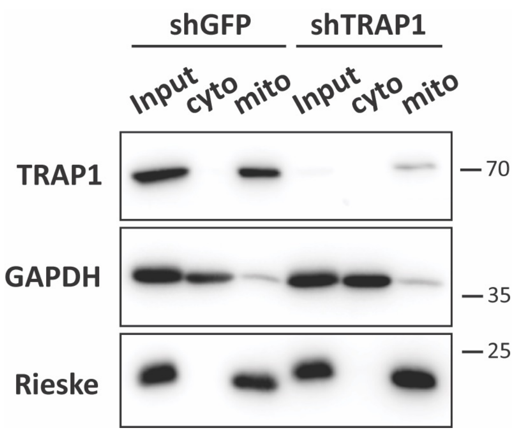

Supplementary Figure 2: Purity of subcellular fractions. Subcelllular fractionation of HeLa cells upon induction of TRAP1-directed shRNAs (shTRAP1) or control shRNAs (shGFP) for 72 hours, obtained as described in the "Materials and methods – Cell fractionation" section. The immunoblot shows the presence of indicated proteins into total lysates (input), cytosolic (cyto) and mitochondrial (mito) fractions. GAPDH has been used as a marker of cytosol, and Rieske protein as marker of mitochondria.

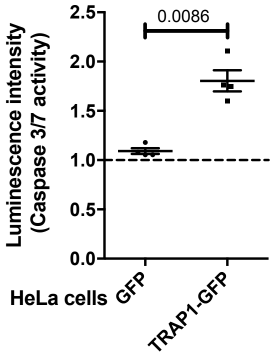

Supplementary Figure 3: Glucose deprivation triggers apoptosis in TRAP1-overexpressing cells. Apoptosis detected by a luminescent caspase 3/7 activity assay in HeLa cells upon complete glucose deprivation (48 hrs) following induction of GFP/TRAP1-GFP (24 hrs). Dashed line indicates the level of the respective control cultured in complete medium. Numbers indicate the statistical significance (two-tailed p-value) based on one-sample t test (n=4).

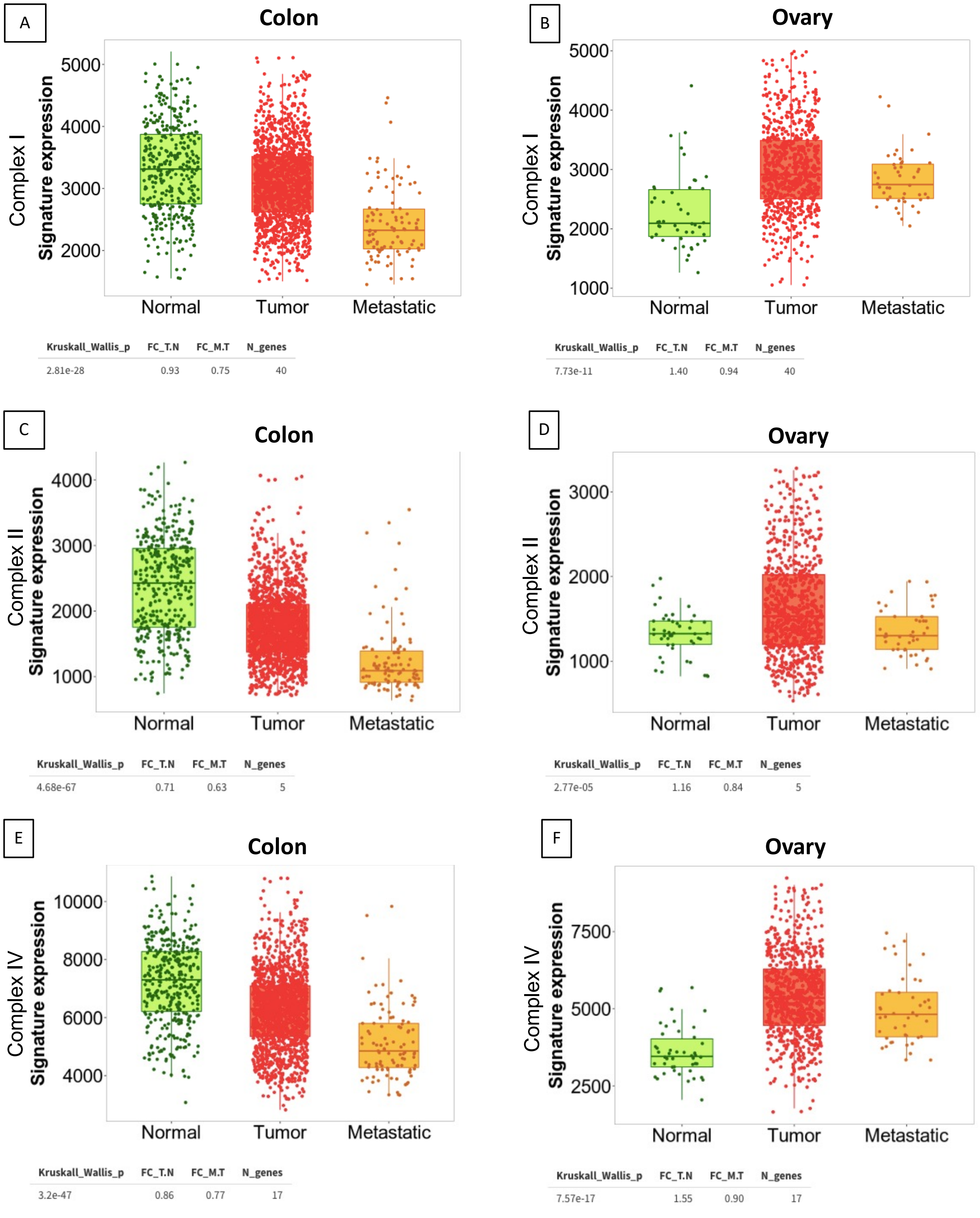

Supplementary Figure 4: Complex I, II and IV are downregulated in colon cancers, and upregulated in ovarian cancers. Boxplot graphs of gene signature analysis based on the expression of complex I (A-B), complex II (C-D) and complex IV (E-F) components and assembly factors in normal tissues compared to tumor tissues and metastatic tumors for colon (A, C, E) and ovary (B, D, F), based on gene chip data obtained using TNMplot. The statistical significance of differential expression was evaluated by the Kruskal-Wallis test.
