# Supplementary material for: Regulation of mitochondrial complex III activity and assembly by TRAP1 in cancer cells
Source: Cancer Cell Int. 2022 Dec 12;22:402. doi: 10.1186/s12935-022-02788-4 (PMC9743594; doi:10.1186/s12935-022-02788-4)
Supplement: Supplementary file 2 — Additional file 2. Original blots [file 12935_2022_2788_MOESM2_ESM.pdf]

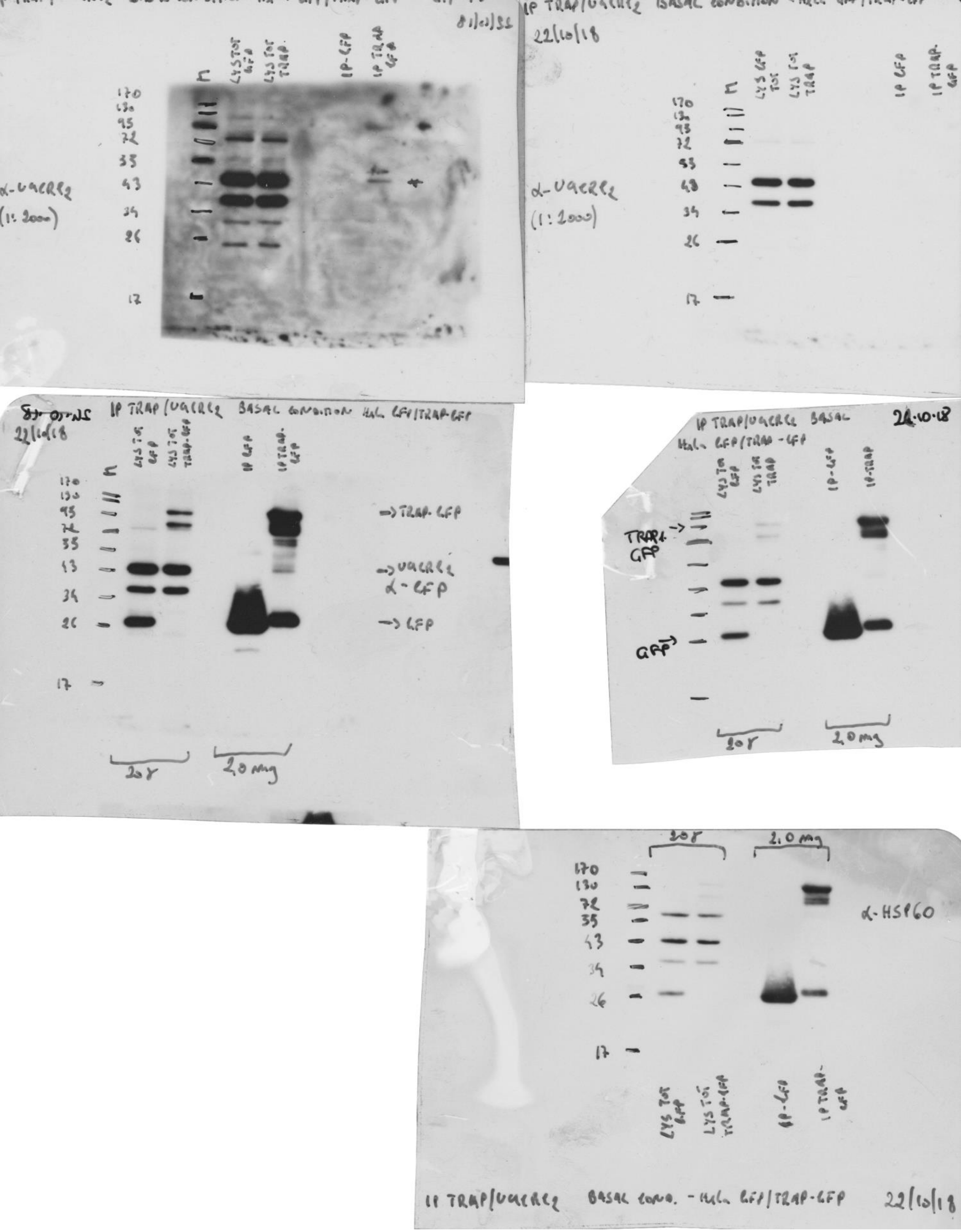

Figure 1 Panel A (left)

Figure 1 Panel A (right)

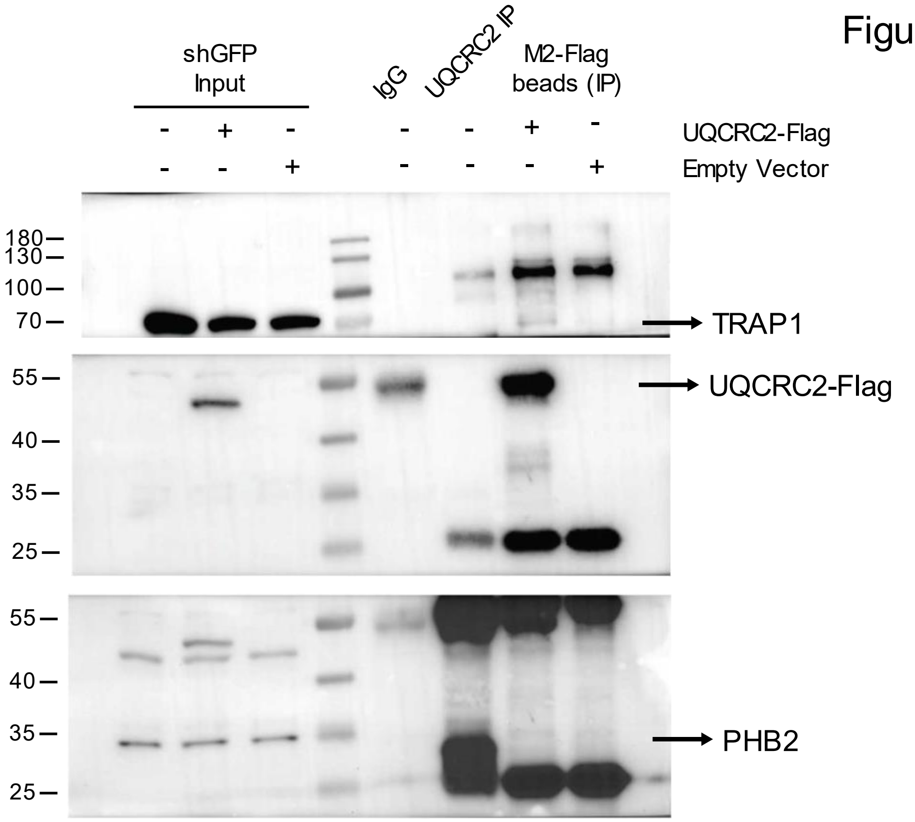

Supplementary Figure 1 Panel A

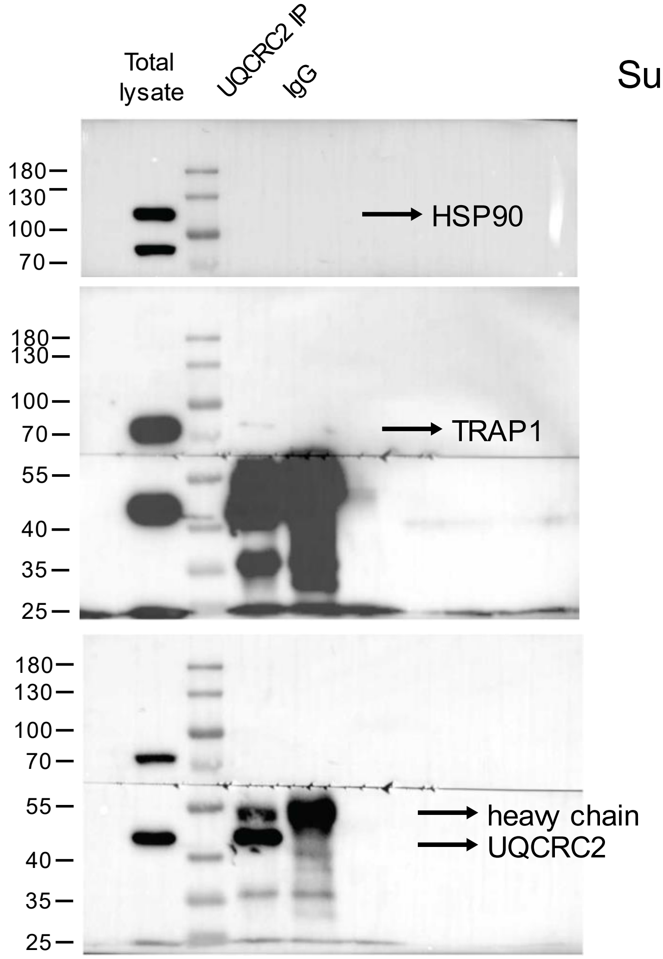

Figure 1 Panel H (right)

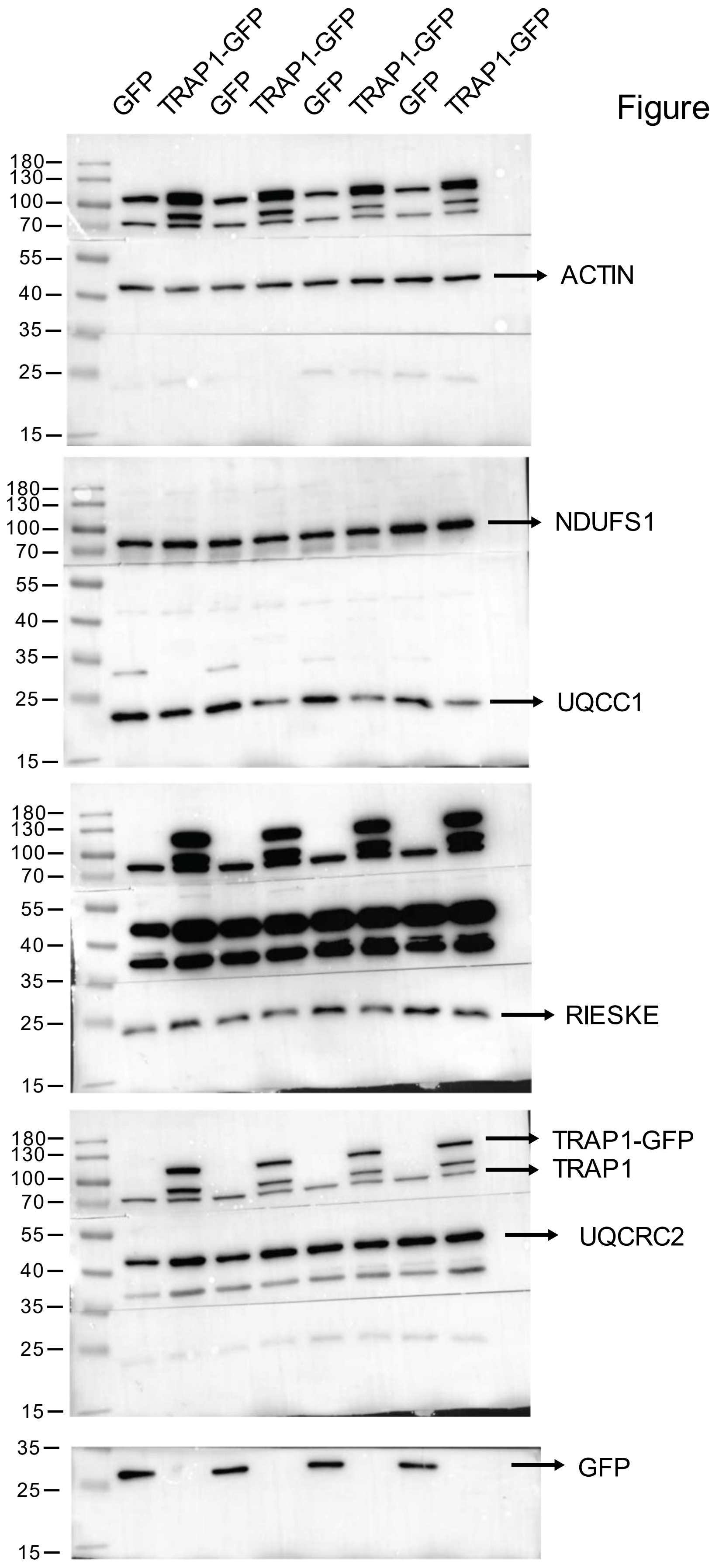

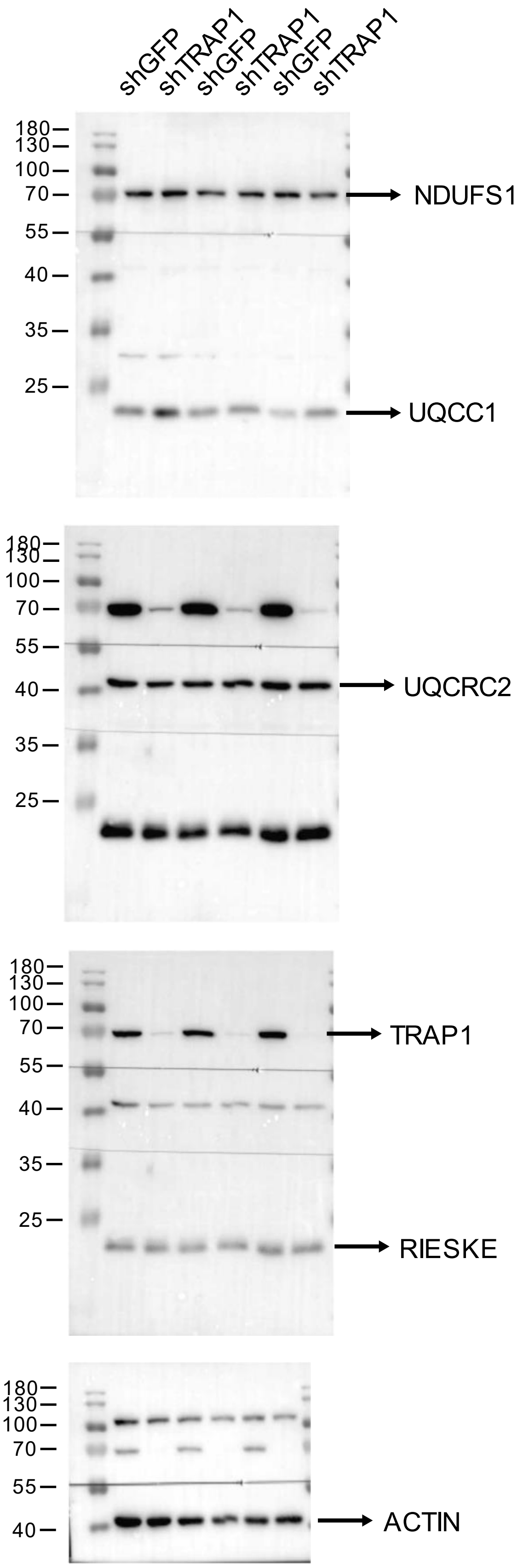

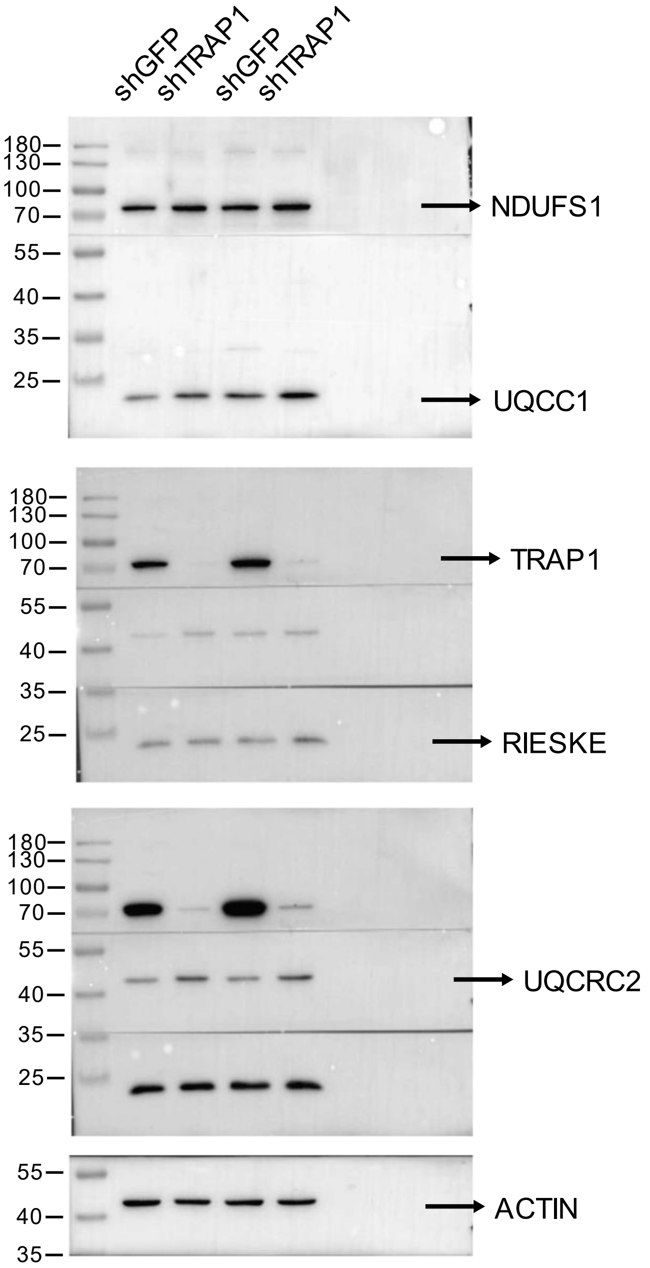

Figure 1 Panel H (left)

Supplementary Figure 2

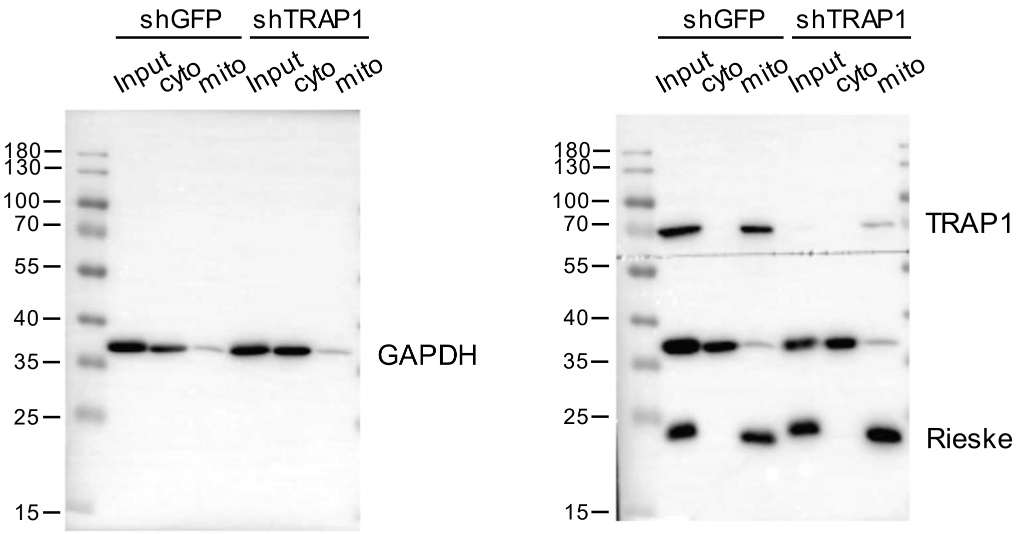

Figure 2 Panel E

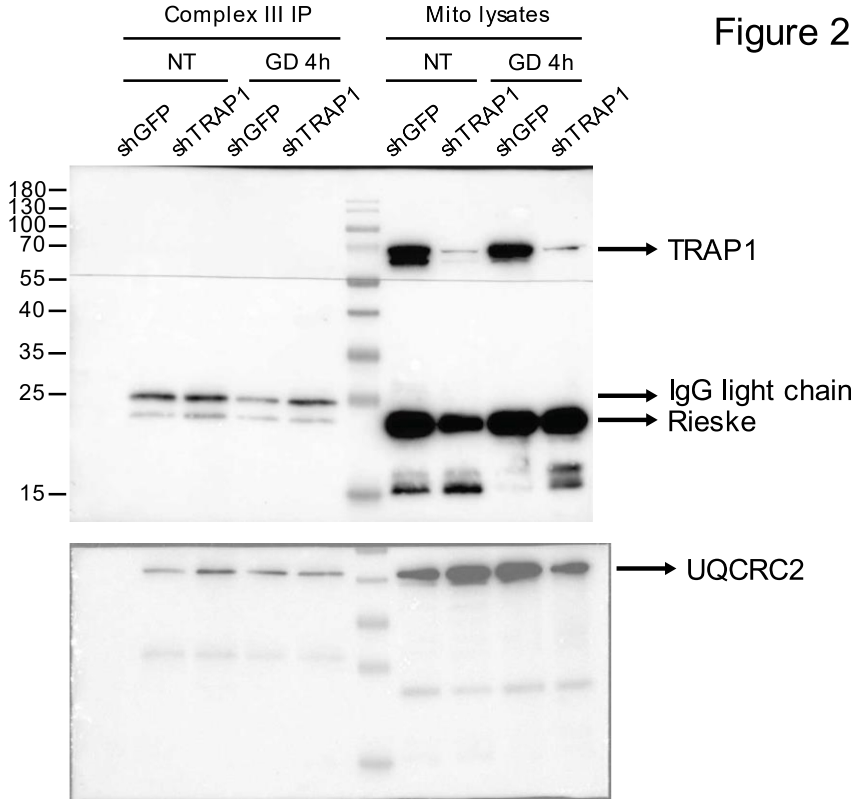

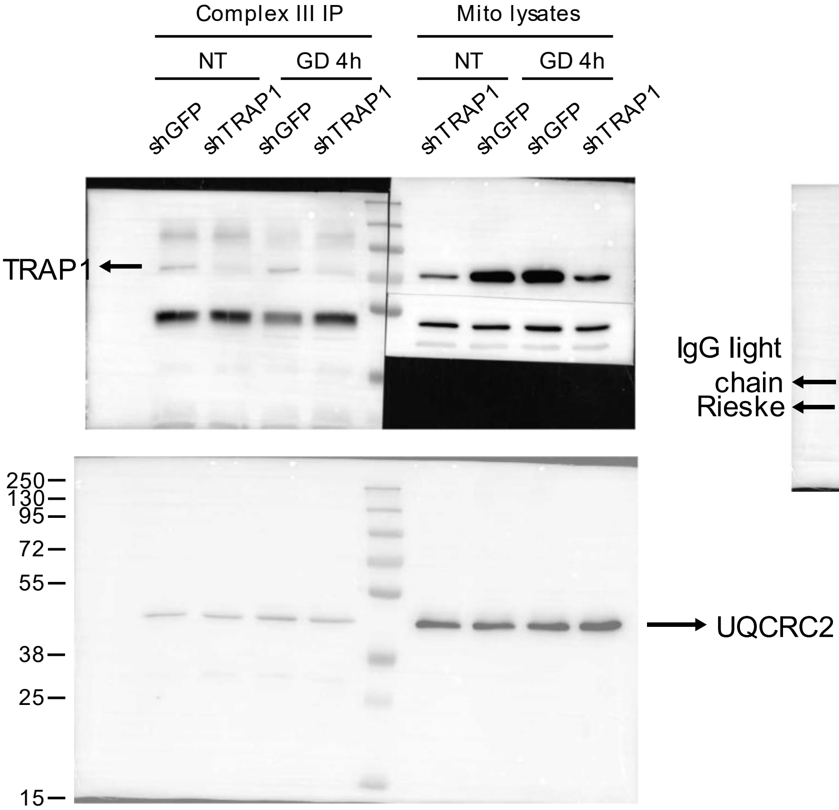

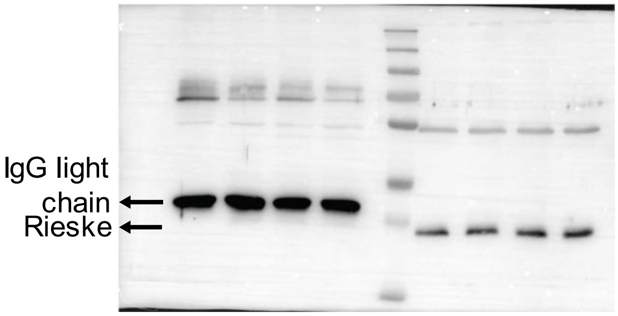

Figure 2 Panel F

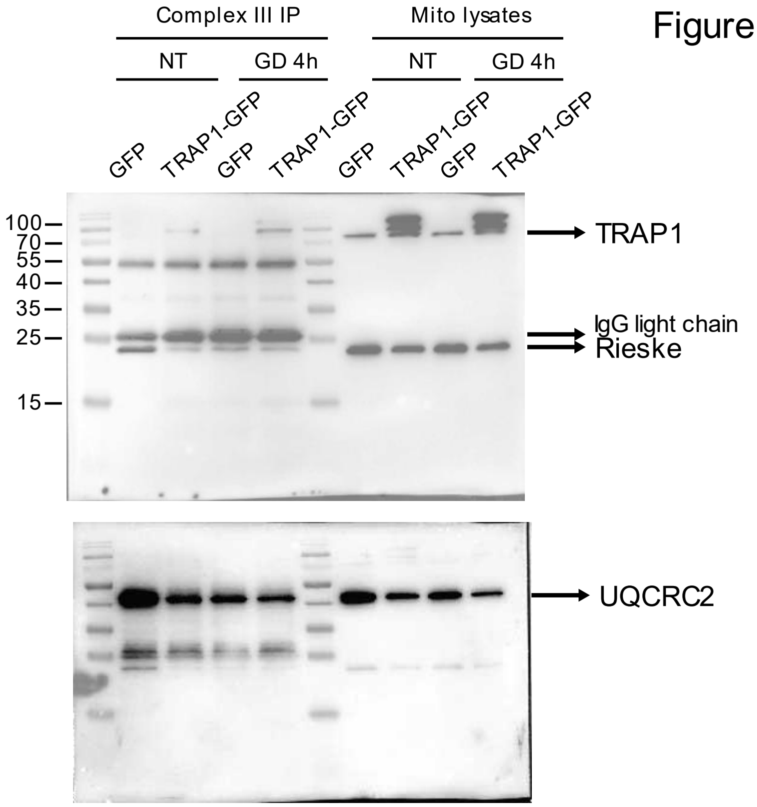

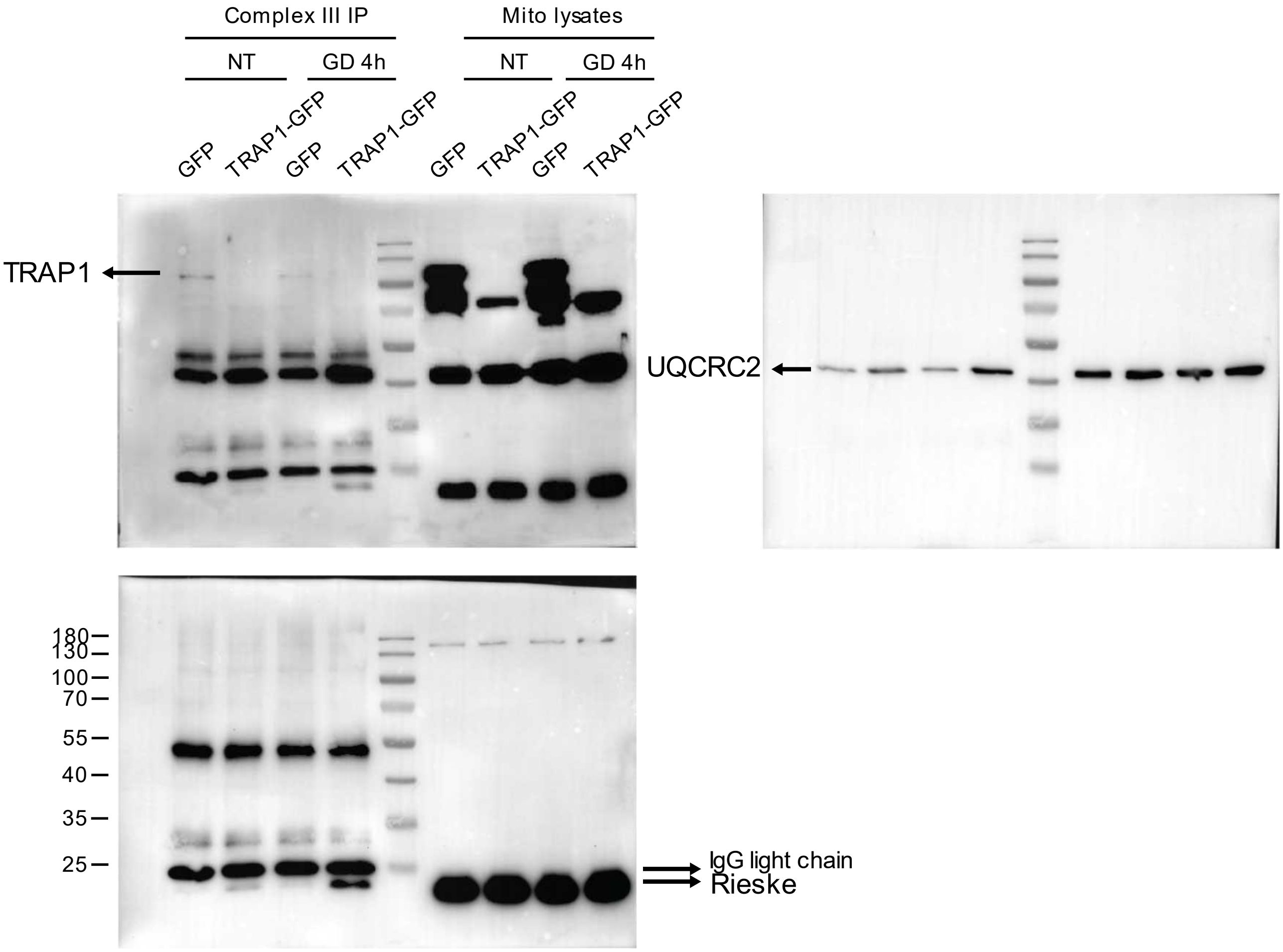

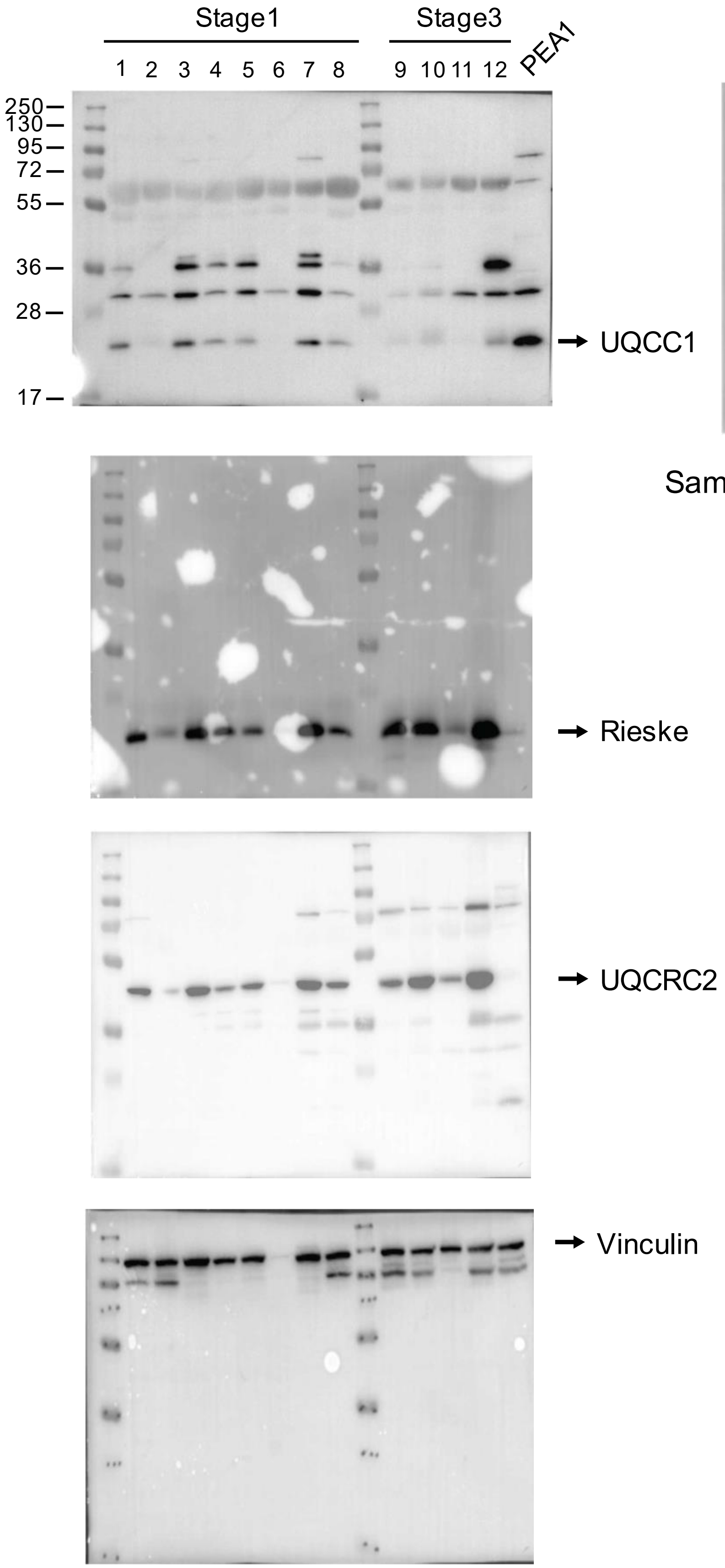

STAIN-FREE blot

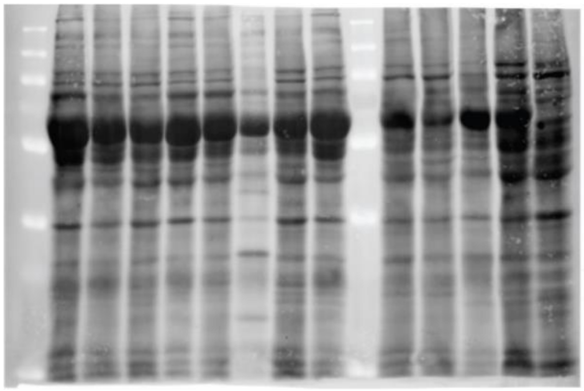

Sample 6 was discarded from the analyses

Figure 4 Panel E-F

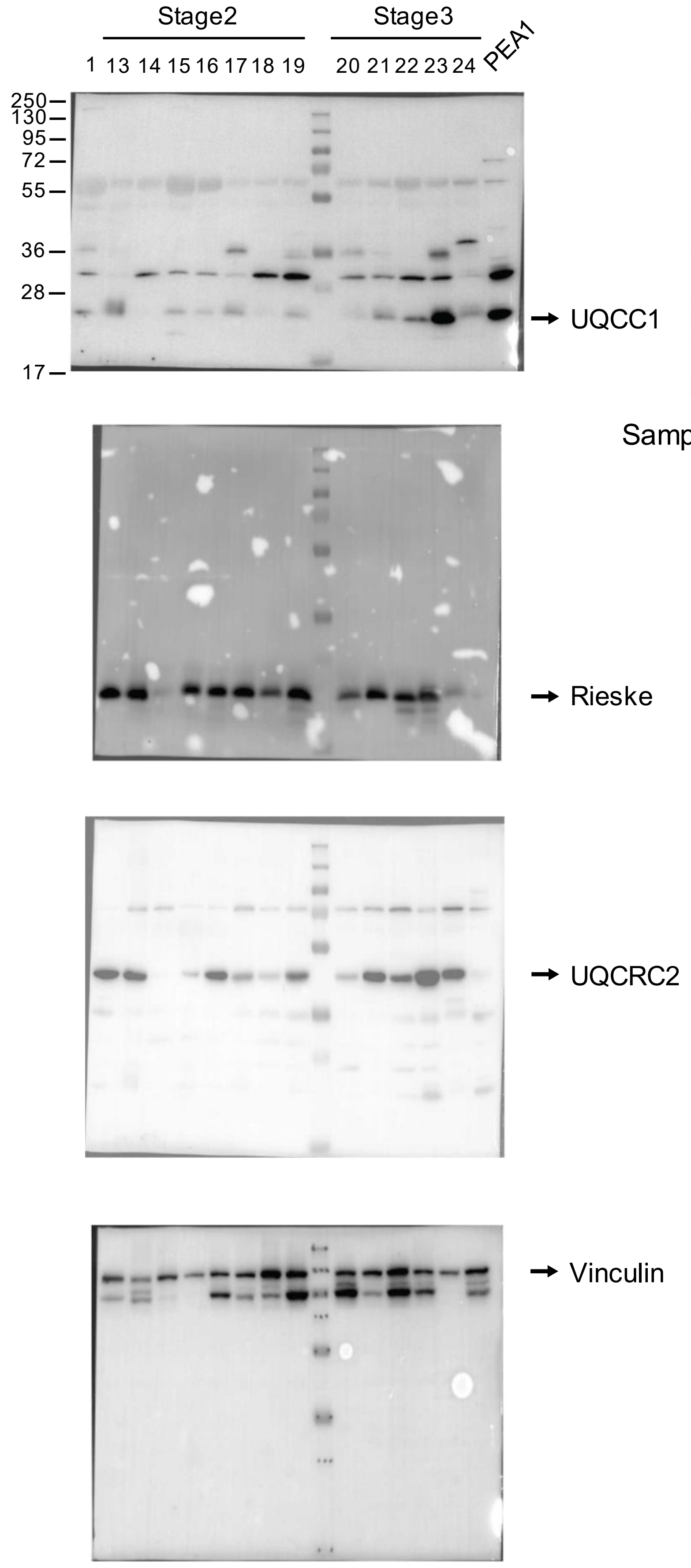

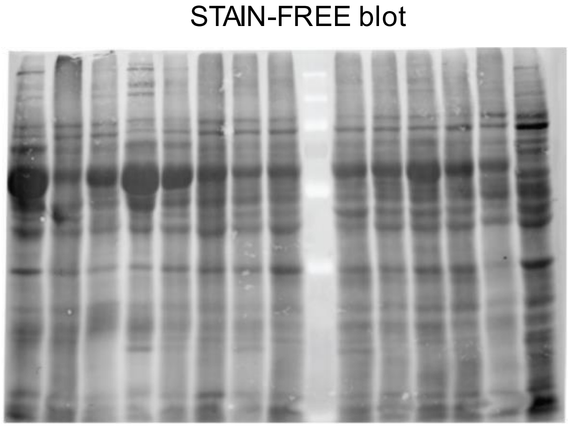

Sample 13 was discarded from the analyses

Figure 4 Panel E-F

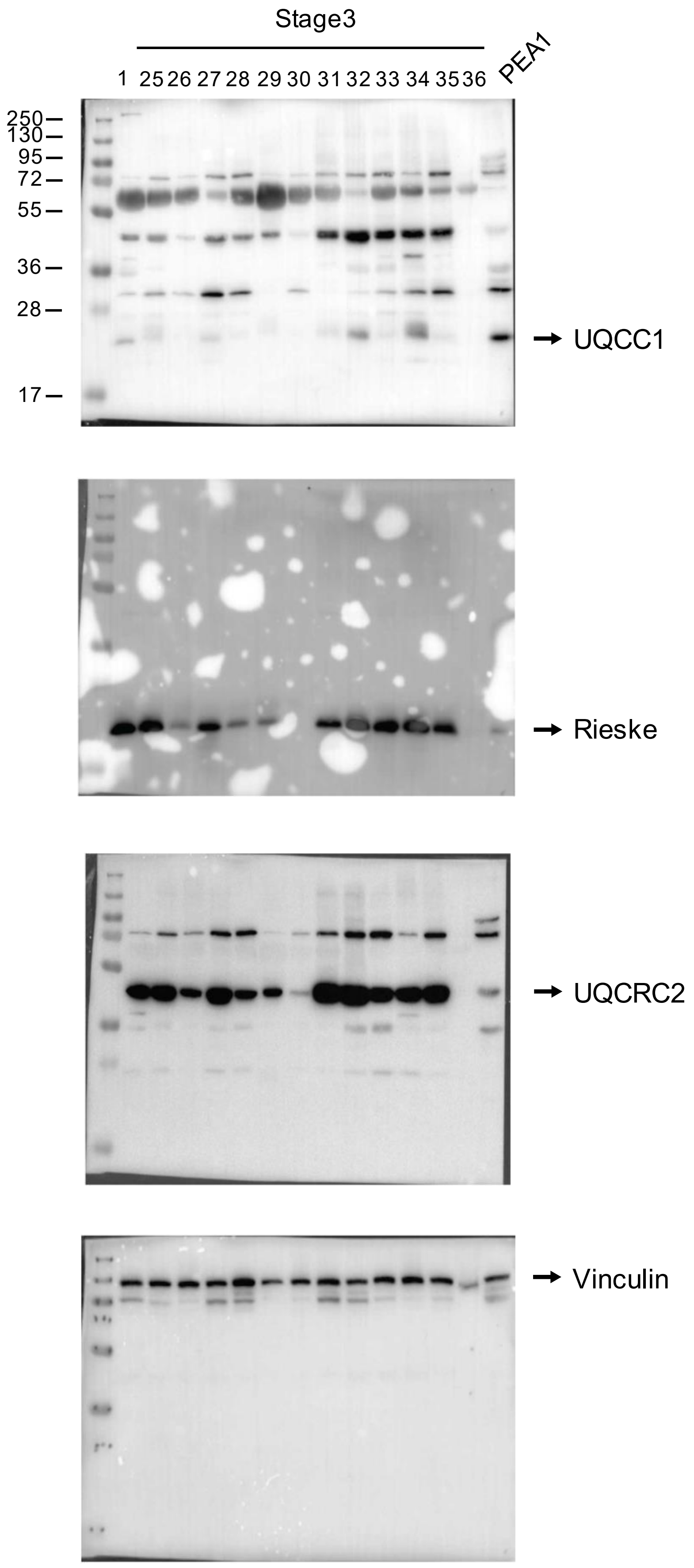

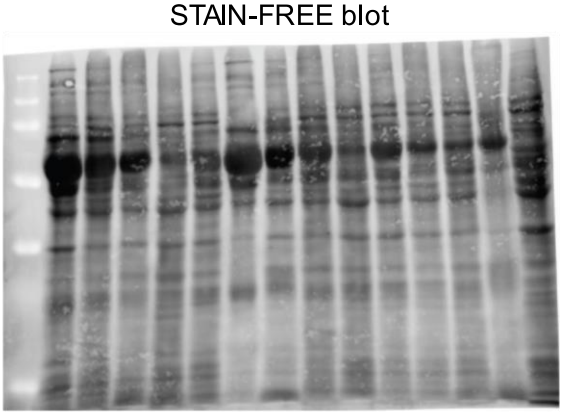

Samples 29 and 36 were discarded from the analyses

Figure 4 Panel E-F

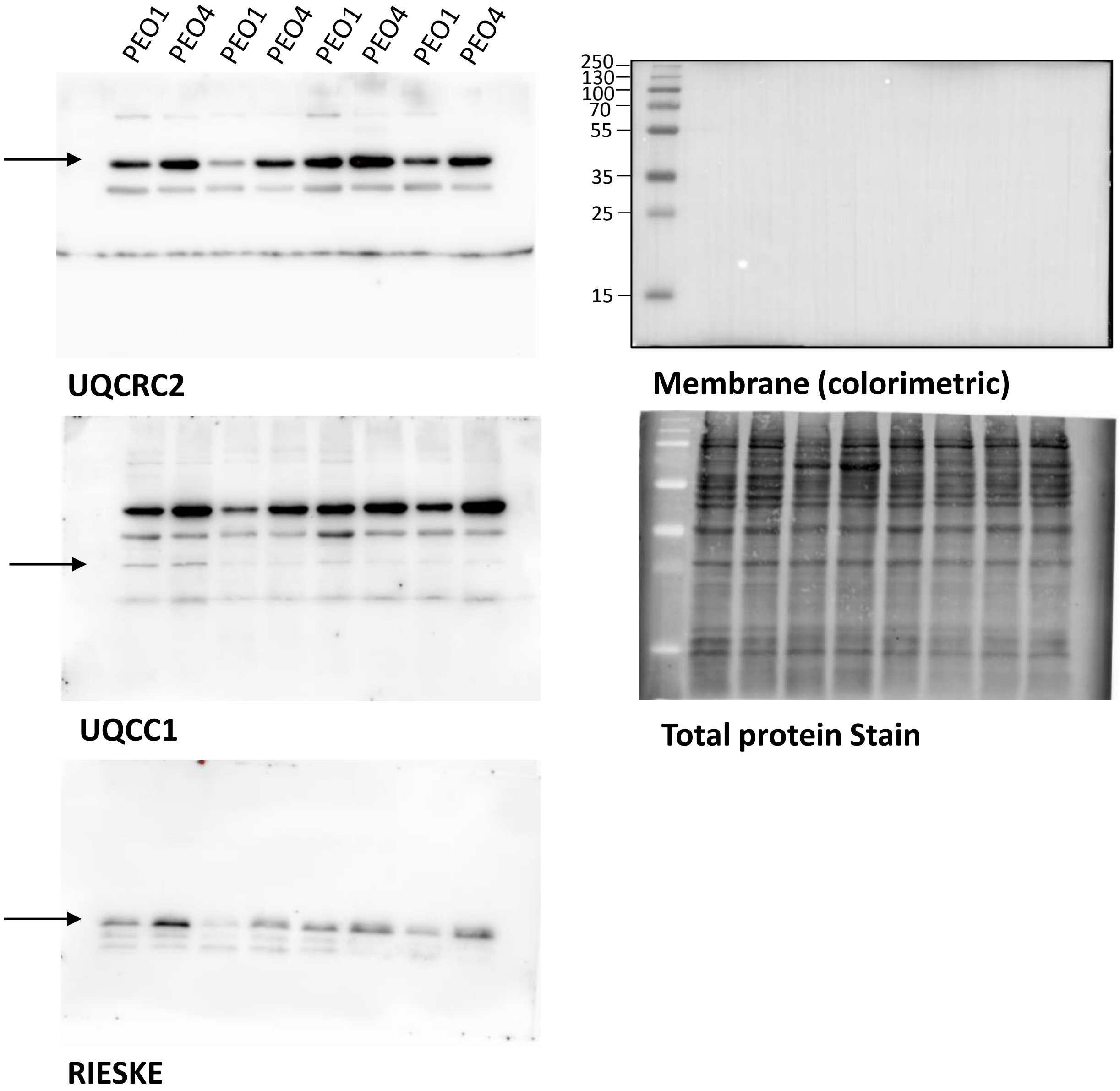

Figure 6 Panel A – left side

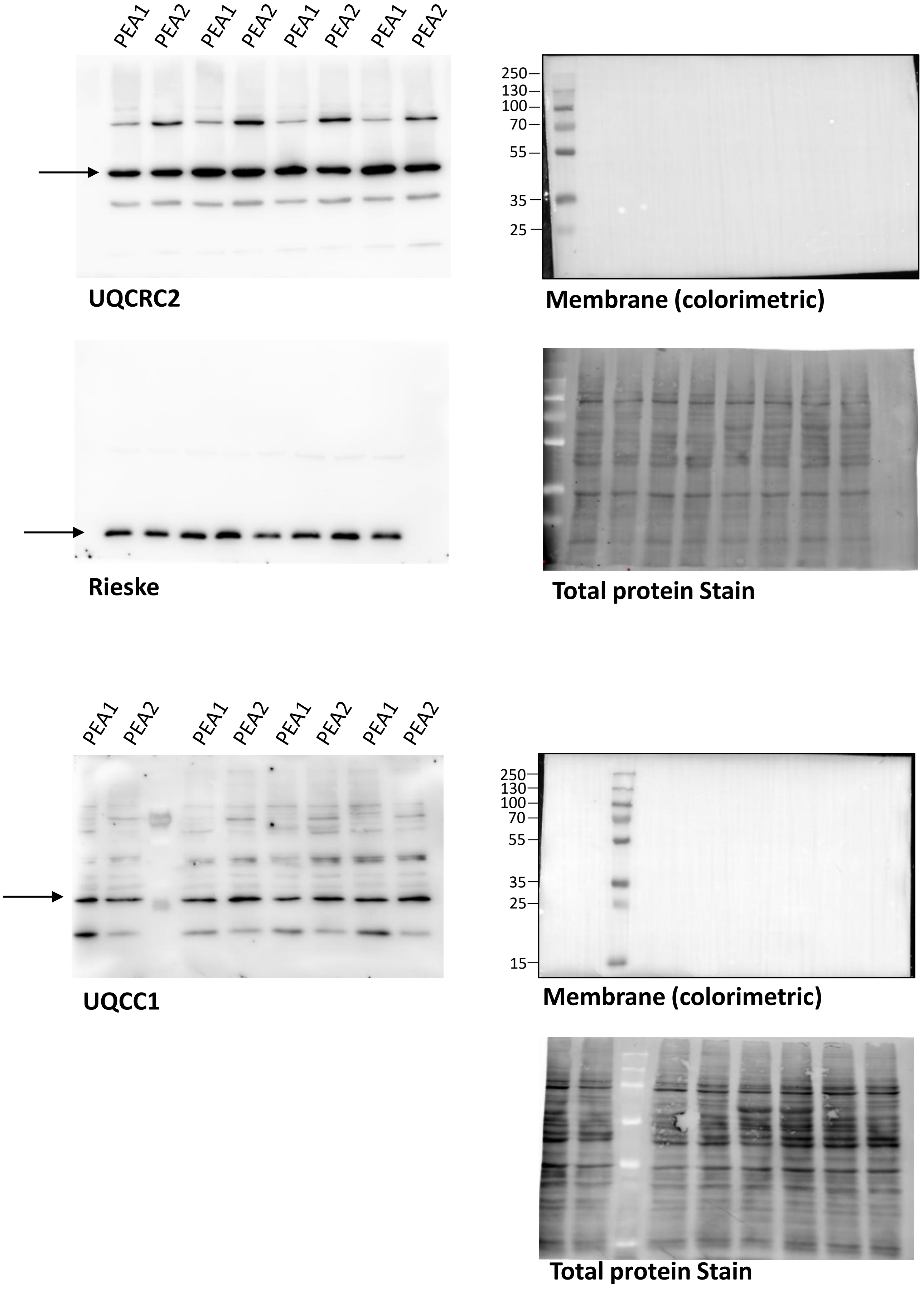

Figure 6 Panel A – right side
